# Ultrasonic synthesis, characterization, DFT and molecular docking of a biocompatible Zn-based MOF as a potential antimicrobial, anti-inflammatory and antitumor agent

**DOI:** 10.1038/s41598-024-71609-7

**Published:** 2024-09-23

**Authors:** Khaled M. Ismail, Fatma B. Rashidi, Safaa S. Hassan

**Affiliations:** Department of Chemistry, Faculty of Science, Cairo University, Giza, Egypt

**Keywords:** Zn-MOF, Biological screening, Antibacterial, Cytotoxicity, Anti-inflammatory, Anti-tumor, DFT, Docking, Biochemistry, Cancer, Chemical biology, Chemistry

## Abstract

Zinc metal–organic frameworks have emerged as promising candidates, demonstrating excellent biological properties stemming from the unique characteristics of MOFs and zinc. In this study, we employed a facile method to synthesize a zinc metal–organic framework **[Zn(IP)(H**_**2**_**O)]** using ultrasound irradiation, with the linker being isophthalic acid (IPA) (1,3-benzene dicarboxylic acid). The parent **Zn-MOF** and two **Ag/Zn-MOF** samples prepared via loading and encapsulation methods were comprehensively characterized using various techniques, including FT-IR, XRD, SEM, TEM, N_2_ adsorption–desorption isotherm, UV–vis spectroscopy and TGA. The parent **Zn-MOF** and two **Ag/Zn-MOF** samples exhibited a broad spectrum of antibacterial effects. Remarkably, genomic DNA of ***P. aeruginosa*** was effectively degraded by **Zn-MOF,** further supporting its potent antibacterial results. The free radical inhibition assay demonstrated a 71.0% inhibition under the influence of **Zn-MOF**. In vitro cytotoxicity activity of **Zn-MOF** against HepG-2 and Caco-2 cell lines revealed differential cytotoxic effects, with higher cytotoxicity against Caco-2 as explored from the IC_50_ values. This cytotoxicity was supported by the high binding affinity of **Zn-MOF** to CT-DNA. Importantly, the non-toxic property of **Zn-MOF** was confirmed through its lack of cytotoxic effects against normal lung cell (Wi-38). The anti-inflammatory treatment of **Zn-MOF** achieved 75.0% efficiency relative to the standard Ibuprofen drug. DFT and docking provided insights into the geometric stability of **Zn-MOF** and its interaction with active amino acids within selected proteins associated with the investigated diseases. Finally, the synthesized **Zn-MOF** shows promise for applications in cancer treatment, chemoprevention, and particularly antibacterial purposes.

## Introduction

The reckless use of antibiotics fosters the proliferation of drug-resistant bacteria, posing a significant challenge to our capacity to manage common infections due to their growing rate of new resistance mechanisms^1^. This resistance problem results from the overuse of antibiotics and mutations in pathogens, together undermining the efficacy of antimicrobial drugs^2,3^. Among the most frequently encountered resistant bacteria are *Escherichia coli*, *Klebsiella pneumoniae*, *Staphylococcus aureus*, and *Streptococcus pneumoniae*^4^.

Different strategies have been proposed to control the development of antibiotic resistance, such as tighter regulation of antibiotic prescriptions and innovative approaches targeting already-resistant bacteria, such as the development of novel antibacterial agents. Various transition metals (Zn, Ag, Cu, etc.) have emerged as promising alternatives to conventional antibacterial agents^5^. Zinc, in particular, plays a crucial role in various biological processes within the human body, serving as an enzymatic cofactor, signaling molecule, and structural element^6^. Additionally, zinc ions contribute to the regulation of Cu/Zn superoxide dismutase (SOD) activity^7^, indirectly modulating intracellular reactive oxygen species (ROS) scavenging. Research indicates a correlation between zinc depletion, decreased Cu/Zn SOD activity, and inflammation^8^, suggesting that exogenous zinc supplementation could potentially alleviate inflammation by restoring SOD activity. Moreover, zinc ions exhibit selective toxicity against cancer cells by inducing DNA damage, ultimately triggering cellular apoptosis. Consequently, zinc is being actively investigated as a therapeutic agent for tumor treatment^9^.

New porous compounds known as metal–organic frameworks (MOFs) have attracted increasing attention in recent years for their potential applications as antibacterial and anticancer materials. Additionally, they serve as a reservoir of metal ions^10^. These compounds exhibit structural characteristics containing cavities in the form of pores and channels, typically possessing high surface area, adjustable shape, and uniform-high pore volumes^11^. MOFs can effectively store metal ions, such as silver (Ag^+^), nickel (Ni^2+^), or copper (Cu^+^ or Cu^2+^) thereby demonstrating superior antimicrobial efficiency. Therefore, MOFs play a crucial role in inhibiting bacterial growth by gradually releasing metal ions through the degradation of framework structures, ensuring sustained antibacterial effects with high durability^12^.

While exposure to large amounts of Zn^2+^ in a short period can be toxic^13^, the relatively stable frame structure of zinc-based MOF enables slow degradation in aqueous environments. This gradual degradation of MOF materials facilitates the long-term release of metal ions, achieving low toxicity levels^14^. Therefore, MOF particles have been shown to exhibit low cytotoxicity, enhancing their application as antibacterial agents relative to the other common disinfectants^1^. It is known that zinc compounds are extensively used in cosmetics and skincare, making them ideal candidates for cancer chemoprevention or as associated agents in clinical treatment^15^. An ideal anticancer agent would exert minimize adverse effects on normal tissues while maximizing its capacity to kill tumor cells and/or inhibit tumor growth^16^. Therefore, MOFs bearing zinc ions hold promise as suitable therapeutic agent for both bacterial and cancer therapies.

Traditionally, MOFs are prepared via room temperature stirring or solvothermal routes at high temperatures with extended reaction times. However, alternative techniques, such as microwaves^17^, electrochemistry^18^ and mechanochemistry^19^ are also employed. Ultrasounds irradiation presents another viable method for MOF synthesis, exploiting its high energy to reduce reaction times and enhance energy efficiency compared to other techniques like electrical heating or microwaves^20^.

A limited number of studies have highlighted the potential of Zn-MOF as potent antibacterial materials. For instance, Diéguez’s et al.^21^ demonstrated the powerful antibacterial activity of MOFs based on zinc skeleton. Building on this foundation, our study aimed to explore the potential of **Zn-MOF** as a drug candidate. On the other hand, other studies have shown that the synthesized starched AgNPs, even at low concentrations, effectively inhibited the growth of various organisms as reported by Jansirani et al.^22^. Furthermore, Darabpour et al.^23^ supported the concept of enhancing antimicrobial activity through the incorporation of AgNPs into **Zn-MOF**. Therefore, we anticipated an enhancement in the antibacterial behavior of the synthesized **Zn-MOF** upon incorporation with AgNPs. AgNPs were introduced through loading (**L-Ag/Zn-MOF)** and encapsulated procedures **(C-Ag/Zn-MOF)**.

Considering the promising biological effects of zinc-based organic frameworks, we aim to report on the biological screening of **Zn-MOF** composed of the isophthalic acid linker, renowned for its biological activity^24^. Ultrasound irradiation is utilized for faster and environmentally friendly synthesis, resulting in reduced reaction time and energy consumption. Furthermore, considering silver’s potential as an antibacterial agent and the observed good surface area of the MOF structures, efforts have been made to evaluate the concurrent antibacterial activity post-silver loading and encapsulation. Bacterial investigation includes the assessment of bacterial DNA damage, while cytotoxicity study extends to several cell lines. Moreover, the cytotoxicity study of **Zn-MOF** involves evaluating double-strand DNA binding and cleavage, along with exploring anti-inflammatory and antioxidant efficacy. Finally, DFT and molecular docking are performed to assess the geometric stability of **Zn-MOF** and its interaction with active amino acids within selected proteins associated with the investigated diseases.

## Experimental

### Materials and physical measurements

All chemicals and solvents, including zinc acetate dihydrate, isophthalic acid (IPA) and ethanol, were of pure analytical grade and obtained commercially from Sigma-Aldrich Company. The synthesized **[Zn(IP)(H**_**2**_**O)]** and two **Ag/Zn-MOF** samples prepared via loading and encapsulation methods were characterized through various techniques using TGA, SEM, TEM, XRD, FTIR, UV–vis spectroscopy and BET surface area analysis.

FT-IR spectra were recorded using a Shimadzu FT-IR spectrometer within the range 400–4000 cm^−1^. Electronic absorption spectra in DMF were measured utilizing an automated UV/Vis–NIR 3101 PC Shimadzu spectrophotometer.

The thermal measurements were performed using a TGA-50H-Shimadzu thermal analyzer under a nitrogen atmosphere, heating from room temperature to 800 ℃ at a rate of 10 ℃ min^−1^. X-ray diffraction analysis involved irradiating the sample with monochromatic X-rays across a range of incident angles. The angles and intensities of the X-ray diffraction were measured using a Bruker LynxEye detector.

The micro-structural morphology was investigated via Scanning electron microscopy (SEM) using a Quanta 250 FEG instrument. The investigation of particle size was conducted using a transmission electron microscope (TEM), specifically a model Jeol JEM 1400 manufactured in Japan, operating at 80 kV. Additionally, specific surface area was obtained utilizing BET analysis with N_2_ adsorption/desorption at 77 K, performed using a gas sorption analyzer (Quantachrome, NOVA, version 11.04). The total pore volume (V_total_) was determined from the amount of adsorbed N_2_ gas at P/P_o._

### Synthesis of [Zn(IP)(H_2_O)] and its silver hybrid forms

Ultrasonic synthesis of **[Zn(IP)(H**_**2**_**O)]** was carried out under ultrasonic irradiation at atmospheric pressure and ambient temperature for 120 min. Specifically, 0.083 g (0.5 mmol) of 1,3- benzene dicarboxylic acid (isophthalic acid, IPA) dissolved in 6 mL ethanol was added to a test tube containing 6 mL of an aqueous solution of 0.109 g (0.5 mmol) zinc acetate dihydrate. Afterward, the test tube was securely placed in the ultrasonic bath. Following irradiation for 120 min, a solid with a white color was isolated.

Ag nanoparticles were prepared according to Jansirani et al. by mixing 20 mL of 1.0 mM AgNO_3_ with 50 mg of starch and adjusting the pH to 11.0 using 0.1 M NaOH^22^. The mixture was stirred for complete dissolution of starch and then subjected to agitation under sonication. To prepare the encapsulated hybrid form**, [Zn(IP)(H**_**2**_**O)]** was synthesized in the presence of the previously prepared AgNPs (2.0 mmol Ag/1.09 g zinc acetate). The loading hybrid form was prepared by mixing AgNPs with **Zn-MOF** (2.0 mmol Ag/1.0 g **Zn-MOF**) in ethanol and then subjected to stirring for 8 h. Subsequently, all samples were washed with a 50% ethanol–water mixture and then dried overnight.

### Well diffusion method

The antibacterial and antifungal effectiveness of synthesized **Zn-MOF** and its hybrid forms, Ag loaded and encapsulated, were evaluated against *Bacillus Subtilis*, *Staphylococcus aureus, Escherichia coli*, *Pseudomonas aeruginosa* and *Candida albicans, Aspergillus Niger* microorganisms. The in vitro antimicrobial activities of the synthesized compounds were assessed using a modified agar well diffusion method^25^. After incubation at 35–37 ℃ for 24–48 h with scanned strains, the diameters of the inhibition zones were measured in millimeters. The growth of the tested strains reached approximately 10^7^ cells/mL using Mueller Hinton media and quantified using a plate counter. A volume of 100 μL of microbial suspension was spread onto agar plates. Subsequently, the volume 10 μL of the tested samples was placed on agar media using blank paper disks.

### *P. aeruginosa* DNA fragmentation

*P. aeruginosa* bacteria were cultured at 10^5^ CFUs/mL in Luria Bertani (LB) broth-rich medium in the presence and absence of the tested compounds at different concentrations ranging from 5 to 25 μg/mL. The cultures were incubated for 4–5 h at 37 ℃ with shaking at 120 rpm in an incubator shaker^26^. Subsequently, the growing bacteria were centrifuged at 8000 rpm for 10 min and washed twice with Phosphate Buffer Solution (PBS) to remove residual media.

Genomic DNA was isolated using the Gene JET Genomic DNA Purification Kit (Thermo Scientific) to assess the genomic DNA integrity. The extracted DNA was evaluated for its integrity using agarose gel electrophoresis. DNA samples were electrophoresed on a 1.0% agarose gel in Tris Boric EDTA (TBE) buffer for 45 min at 90 V. The gel was then stained with ethidium bromide (EtBr), and an image was captured using a mobile camera.

### Antioxidant and antitumor study

Free radical scavenging activity was measured by 1,1-diphenyl-2-picryl hydrazyl (DPPH) assay. Initially, a 0.1 mM solution of DPPH in ethanol was prepared. Subsequently, 1 mL of this solution was added to 3 mL of different concentrations (3.9, 7.8, 15.62, 31.25, 62.5, 125, 250, 500, 1000 μg/mL) of **[Zn(IP)(H**_**2**_**O)]** in DMSO. The mixture was vigorously shaken and allowed to stand at room temperature for 30 min, then absorbance was measured at 517 nm using a spectrophotometer. Ascorbic acid was used as the reference standard compound, and the experiment was done in triplicate^27^. The IC_50_ value of the tested sample was calculated. The percentage DPPH scavenging effect was calculated using the following equation:

$$\text{DPPH scavenging effect }\left(\%\right)= \frac{{A}_{o} -{A}_{S}}{{A}_{o}} x 100,$$

where A_0_ represents the absorbance of the control reaction and A_S_ represents the absorbance in the presence of a test or standard sample.

The cytotoxic effect of **[Zn(IP)(H**_**2**_**O)]** was determined by using a 3-(4,5-dimethylthiazolyl)2,5-diphenyl-tetrazolium bromide (MTT) assay^28^. Initially, each cell line, including HepG2, Caco2 and Wi-38 was inoculated in a flat-bottomed 96-well plate at a density of 10^4^ cells/100 µL complete RPMI medium/well and allowed to develop a complete monolayer sheet overnight. After 24 h, the cell monolayer sheet was washed twice with wash media and treated with a two-fold dilution series of the tested samples ranging from 0.0 to 1000 µg/mL. The dilutions were achieved by dissolving the proper weight of the tested sample in 1 mL of PRMI medium containing 2% fetal bovine serum (FBS). Subsequently, 100 µL of each diluted sample was loaded in different cultured wells, and the experiment performed in triplicate. Three wells containing inoculated cells received culture medium and served as untreated groups. The plate was then incubated for 24 h in a humidified incubator at 37 ℃ with 5% CO_2_. After that, cells were examined under an inverted microscope to observe any signs of toxicity induced by **[Zn(IP)(H**_**2**_**O)]** on each cancer cell line. MTT solution (BIO BASIC CANADA INC) (20 µL of 5 mg/mL PBS) was added to each well, and the plate was shaken at 150 rpm for 5 min to ensure thorough mixing. Subsequently, the plate was returned to the incubator to allow MTT to metabolize and appear as purple formazan crystals. After incubation, the medium was dumped off and the formazan crystals were dissolved in 200 µL of DMSO by shaking the plate on the shaking table for 5 min at 150 rpm. The optical densities (OD_s_) of the dissolved formazan crystals were determined spectrophotometrically at 560 nm, with background subtraction at 620 nm. The results were expressed in terms of the concentration required to inhibit cell growth by 50% relative to untreated cells (IC_50_).

$$Cell-viability=\frac{OD treated-cells}{OD untreated-cells}\times 100.$$

### CT-DNA binding

The DNA binding ability with **[Zn(IP)(H**_**2**_**O)]** was evaluated by monitoring changes in DNA gel electrophoresis-induced binding by gradual increasing concentration of **[Zn(IP)(H**_**2**_**O)]**. The concentration range was 5–25 µg/mL in a buffer solution containing 20 mM Tris HCl and 50 mM NaCl at pH 7.3, with a constant concentration of CT-DNA (25 µg/mL). The incubation time was carried out for 2 h at 37 ℃. For the gel electrophoresis, a 1% w/v agarose gel was prepared in TBE buffer (45 mM Tris HCl, 45 mM of boric acid, and 1 mM EDTA, pH 7.3). The binding effects of DNA were reflected in both DNA migration pattern and intensity on the gel electrophoresis technique. After the incubation period, the reaction mixture was stopped by adding 6 × gel loading dye and electrophoresis was performed at 60 V for 45 min. Then the gel was stained with ethidium bromide (EB) at room temperature for 5 min and visualization of the gel was achieved using a UV-transilluminator, and the gel image was captured using a mobile camera.

### DNA cleavage

The impact of the prepared **[Zn(IP)(H**_**2**_**O)]** on the DNA cleavage of PBR322 (100 ng) was evaluated using concentrations ranging from 5 to 20 μg/mL in a final reaction mixture of 10 μL in the presence of control. Each reaction was then incubated in darkness at 37 ℃ for 1 h. To halt the reaction, 6 × loading dye was added, and the samples were monitored by running them on 1.0% agarose gel in TBE buffer at 70 V for 45 s, then the gel was stained by EB and photographed using mobile camera.

### Denaturation inhibition measurements of bovine serum albumin

In the protein denaturation inhibitor screening, a modified method of Gambhire et al. was employed^29^. In a reaction volume of 5 mL, consisting of 4.78 mL PBS buffer (pH 7.4) and 200 µL of 1.0% of Bovine Serum Albumin (BSA), **[Zn(IP)(H**_**2**_**O)]** at a concentration of 10 µg/mL was used. The reaction solution was incubated at 37 ℃ for 15 min, followed by heating at 70 ℃ for 5 min, then the reaction mixture allowed to cool, and the developed turbidity was estimated spectrophotometrically at 660 nm. The control sample comprised a PBS and BSA in the absence of **[Zn(IP)(H**_**2**_**O)]**. The percentage of inhibition of denatured protein was calculated using the formula:

$$\text{Percentage of inhibition}=\left(1- \frac{{A}_{2}}{{A}_{1}}\right)x 100,$$

where A_1_ and A_2_ represent the absorbance values of the control sample and the tested **[Zn(IP)(H**_**2**_**O)],** respectively.

### Computational study

The calculations were performed using Gaussian 09 revision A.02^30^ employing the DFT/B3LYP method. The standard basis sets used were 6-311G for IPA and LANL2DZ for **Zn-MOF**. Molecular docking was carried out using MOE 2008 (Molecular Operating Environment). The protein crystal structure utilized in the docking studies was obtained from the Protein Data Bank (PDB). Additionally, Adsorption Locator within Material Studio 4.3 software was utilized for investigating individual systems of various crystal planes, enabling the identification of low-energy adsorption configurations.

## Results and discussion

### Characterization

#### FTIR spectra

**Fig. 1:**
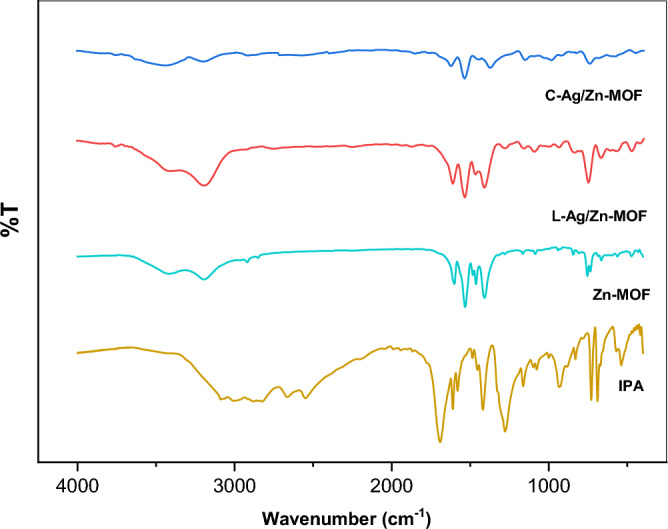
IR spectra of isophthalic acid (IPA), Zn-MOF and Ag/Zn-MOF.

The FTIR spectrum of the synthesized **Zn-MOF** sample revealed characteristic features (Fig. 1**)**. The O – H stretching modes of water molecules typically appear above 3000 cm^−1^. The FTIR spectrum of **Zn-MOF** exhibits two bands at 3415 and 3192 cm^−1^, which are assigned to the antisymmetric and symmetric OH stretching modes, respectively, of coordinated water molecules^31^. The spectrum of IPA exhibits a strong and broad band centered around 3000 cm^−1^ which corresponding to (O–H) vibrations of hydrogen bonds. This is typical for carboxylic acids, which usually exist as hydrogen-bonded dimers^32^. The absence of an absorption band around 3000 cm^−1^ in the spectrum of the **Zn-MOF** confirmed the full deprotonation of the IPA linker during the formation of the **Zn-MOF**.

The IR bands corresponding to the ν(C = O) and ν(C – O) modes of the IPA are reported at ca. 1690 and 1280 cm^−1^, respectively^33,34^. However, in the **Zn-MOF**, these absorption bands are replaced by the ν(COO^−^) of carboxylates with C – O bond^31^. Moreover, the C – O stretching modes of the carboxylate groups are identified by the presence of antisymmetric and symmetric vibrations, ν_as_(COO^−^) at ca. 1600, 1532 and ν_s_(COO^−^) at ca. 1462, 1408 cm^−1^, respectively. The difference in wavenumbers between these two frequencies {Δ = ν_as_(COO^−^) − ν_s_(COO^−^)} provides information on the binding mode of the carboxylate groups to zinc metal centers. Typically, values of Δν_exp_ above 180 cm^−1^ are indicative of monodentate coordination, whereas values of Δν_exp_ below 120 cm^−1^ usually suggest chelating or bridging carboxylate groups^31,35^. For **Zn-MOF**, the experimental values of Δν_exp_ are 192 and 70 cm^−1^, corresponding to monodentate and bridging (or chelating) binding mode, respectively. However, it is essential to note that these correlations are empirical and cannot be used for a definitive structure.

Moreover, the O – H modes of IPA are replaced by M – O modes, observed at low frequencies^31^, with bands appearing at 472 and 469 cm^−1^ assigned to Zn – O^36,37^. These analyses suggested that IPA was successfully converted to **Zn-MOF,** which presents one fourfold-coordinated zinc ion with two bridging oxygen and one monodentate from carboxylate linker, along with one additional coordinated water molecule, i.e. **[Zn(IP)(H**_**2**_**O)]**.

The FT-IR spectra of the Ag/Zn-MOF did not show significant differences compared to the spectrum of Zn-MOF alone, with changes primarily observed in peak intensities rather than positions. In general, the similarity observed suggests that the coordination states of the components remain unchanged following silver incorporation^38^.

#### Uv–vis spectra

**Fig. 2:**
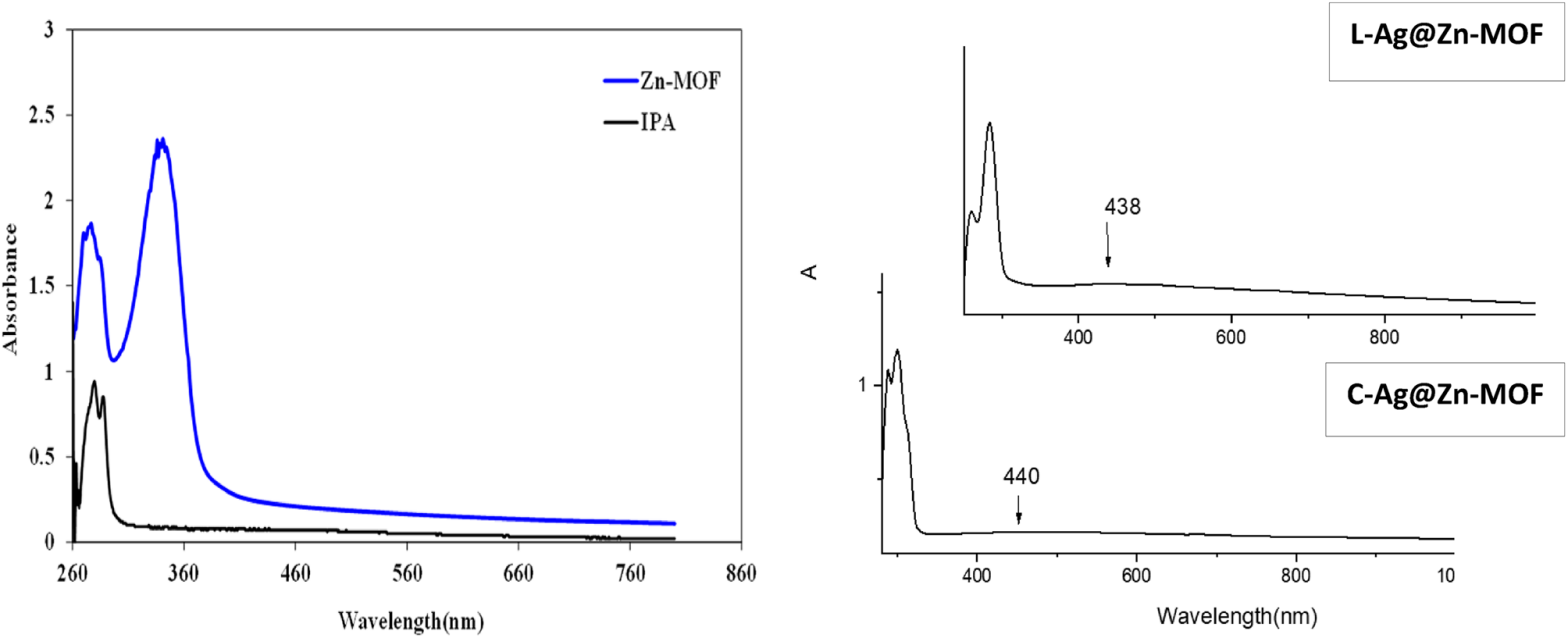
UV–Vis spectra of isophthalic acid (IPA), Zn-MOF and Ag/Zn-MOF.

The transitions corresponding to the bands of the IPA ligand and its **Zn-MOF** were represented in Fig. 2. IPA showed transitions at 279.0 and 288.0 nm, which can be assigned to π-π* transitions, specifically involving the aromatic part, and n-π* transitions of the non-bonding electrons of (C = O), respectively. Upon bonding formation, the transition spectrum of the MOF observed at 277.0 and 341.0 nm. Since Zn(II) ions have a d^10^ electron configuration and are difficult to oxidize or reduce, the transitions observed are probably attributed to intraligand π - π* transitions, perturbed by metal coordination^39^. In Zn(II) carboxylate-based compounds, the HOMO and LUMO are likely the π-bonding orbital from the aromatic rings and the Zn – O (carboxylate) π*-antibonding orbital, respectively, often localized on the metal centers^37–40^. The d^10^ configuration and its diamagnetic properties Zn(II) ions suggest a possible tetrahedral geometry.

The aqueous silver ions were reduced to silver nanoparticles (AgNPs), resulting in a brownish-yellow color, indicative of AgNPs formation, as illustrated in the experimental section. The formation and stability of these nanoparticles were examined using UV–Vis spectrophotometry, with the maximum absorbance observed at 438 nm, consistent with previous findings^22,41^.

The successful attachment and incorporation of the AgNPs onto the **Zn-MOF** are highlighted in the UV–Vis experiments, as seen in Fig. 2. The UV–vis spectra of **Ag/Zn-MOF** samples retained the characteristic features of **Zn-MOF**, exhibiting a narrow and sharp excitation wavelength in the range of 283–311 nm, while the presence of AgNPs was evident at 438 and 440 nm for **L-Ag/Zn-MOF** and **C-Ag/Zn-MOF**, respectively.

#### Thermal analysis

**Fig. 3:**
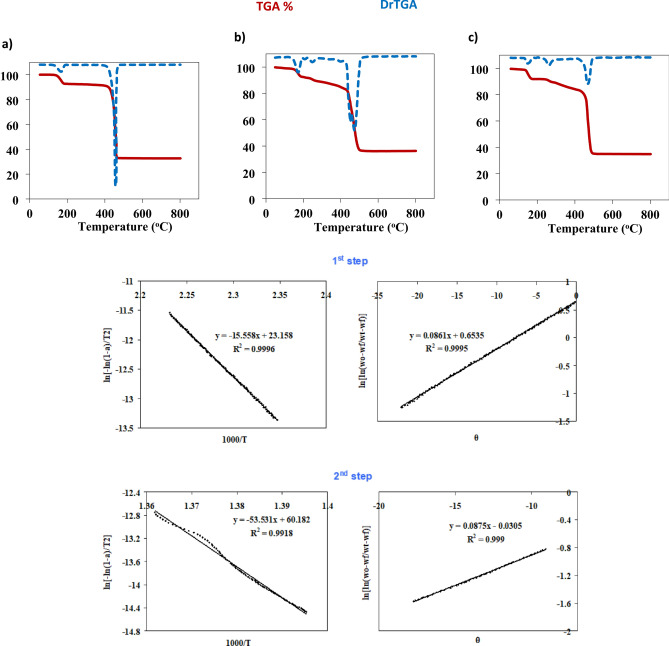
TG curves of Zn-MOF, L-Ag/Zn-MOF and C-Ag/Zn-MOF, respectively, and Coats–Redfern Horowitz-Metzger plots for the decomposition steps of Zn-MOF.

Figure 3 shows the thermogram of the synthesized ultrasonic-assisted **[Zn(IP)(H**_**2**_**O)]** sample under the optimized inert condition. Thermal behavior of the compound is characterized by two distinct regions. In the first region, observed at 170.0 ℃, there is a loss of the coordinated water molecule directly bonded to the zinc ion, resulting in a reduction in initial mass with a loss percentage of 7.66% (calc. = 7.27%). The second step involves the loss of the organic moiety (isophthalate) at 457.3 ℃, with a weight drop of 59.68% (calc. = 59.84%). This step leaves behind ZnO residue, with a mass of 32.66% (calc. = 32.88%).

It’s worth noting that a coordinated IP in **[Zn(IP)(H**_**2**_**O)]** remains stable up to 457.3 ℃, whereas IPA sublimes at 300 ℃^42^. Moreover, the excellent agreement between the observed and calculated mass loss confirms the successful preparation of **[Zn(IP)(H**_**2**_**O)]**.

For the two **Ag/Zn-MOF** samples, a similar decomposition behavior to **[Zn(IP)(H**_**2**_**O)]** was observed, as seen in Fig. 3. The decomposition occurred in two main weight loss steps. For **L-Ag/Zn-MOF** and **C-Ag/Zn-MOF**, the second weight loss step occurred at relatively higher temperatures (474 and 467 ℃, respectively) compared to **Zn-MOF**, indicating the improved thermo-stability of the MOF after AgNPs incorporation. The presence of silver nanoparticles in the structures leads to higher residual percentage values compared to pristine MOF, as the final residues comprise not only zinc oxide but also silver metal. This results in 36.1 and 35.0% residual precipitate for **L-Ag/Zn-MOF** and **C-Ag/Zn-MOF**, respectively. The estimated silver precipitate accounts for 3.4 and 2.3% relative to **L-Ag/Zn-MOF** and **C-Ag/Zn-MOF**, respectively.

Coats–Redfern (CR) and Horowitz-Metzger methods were used to evaluate the kinetic and thermodynamic parameters (ΔE, ΔH, ΔS, and ΔG)^40^, as seen in Fig. 3 and summarized in Table 1. The high values of the activation energy (ΔE*) can be related to the stability of the investigated **Zn-MOF**. The activation entropies ΔS* have small positive values which may indicate a small change in the ordered state of the activated compounds and the reactants^43^. Although the change is small, the positive value suggests an increase in disorder during the reaction. The positive values of ΔG* indicate the non-spontaneity of the processes. This implies that the processes are not favored in terms of spontaneity and require an input of energy to proceed.

**Table 1:** The activation parameters* for the decomposition of Zn-MOF using Coats-Redfern and Horowitz-Metzger methods respectively.

Compound	Step	R	ΔE* (kJ/mol)	ΔH* (kJ/mol)	ΔS* (kJ/mol.K)	ΔG* (kJ/mol)
Zn-MOF	1^st^	0.999793185 0.999744668	129.35 99.00	126.26 95.91	0.0452 0.0181	109.45 89.16
	2^nd^	0.995914268 0.9995097	445.06 100.73	441.96 97.63	0.3633 0.0229	306.83 89.11

#### X-Ray diffraction, SEM and physisorption isotherms

**Fig. 4:**
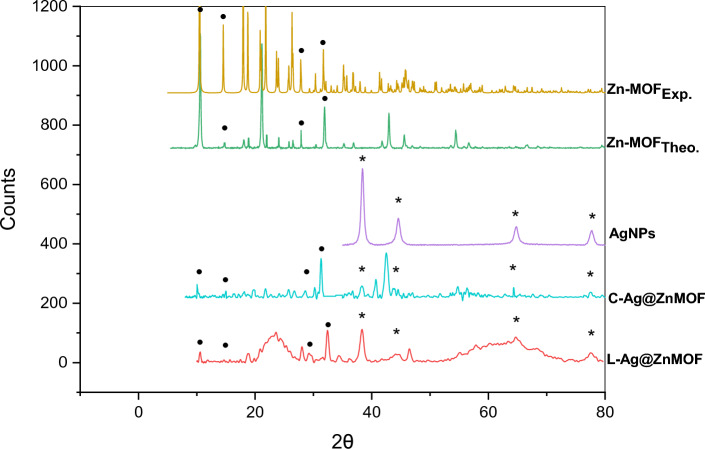
XRD results of as-synthesized [Zn(IP)(H_2_O)] compared with the simulated one and Ag/Zn-MOF.

Figure 4 illustrates the XRD pattern of the prepared **[Zn(IP)(H**_**2**_**O)]**. The sharp peaks observed in the pattern indicate its inherent crystallinity. The compatibility between the experimentally observed XRD pattern of the prepared **Zn-MOF** and the theoretical XRD pattern of the previously reported single crystal of [Zn(C_8_H_4_O_4_)(H_2_O)]_n_ was observed in Fig. 4^44^. The XRD results of the MOF demonstrate diffraction peaks at 2θ = 10.46°, 14.64°, 17.96°, 18.74°, 21.02°, 21.87°, 23.87°, 26.56°, 31.77°, 42.77°, 45.42° which are ascribed to (002), (010), (012), (111), (200), (201), (104), (211), (006), (008), (217) reflections, respectively. Table 2 displays a comparison between the theoretical and experimental peaks positions of the prepared **[Zn(IP)(H**_**2**_**O)]**.

**Table 2:** The sharp peaks of the theoretical and experimental values of the prepared Zn-MOF.

Reflections	2θ (Exp.)	2θ (Calc.)
002	10.46	10.45
010	14.64	14.59
012	17.96	17.92
111	18.74	18.72
200	21.02	21.04
201	21.87	21.82
104	23.87	23.68
211	26.56	26.28
006	31.77	31.73
008	42.77	42.83
217	45.42	45.86

The XRD characterization of the two **Ag/Zn-MOF** samples revealed that their crystal structures remained intact, with the main peaks observed at the same positions (Fig. 4**)**. However, slight differences in peaks positions and intensities were observed, which could be due to changes in their morphology during synthesis. The characteristic peaks in the XRD pattern of **Ag/Zn-MOF** observed at 38.0°, 44.2°, 64.4°, and 77.5° corresponding to (111), (200), (220) and (330) planes of Ag, respectively (JCPDS file 04-0783).

**Fig. 5:**
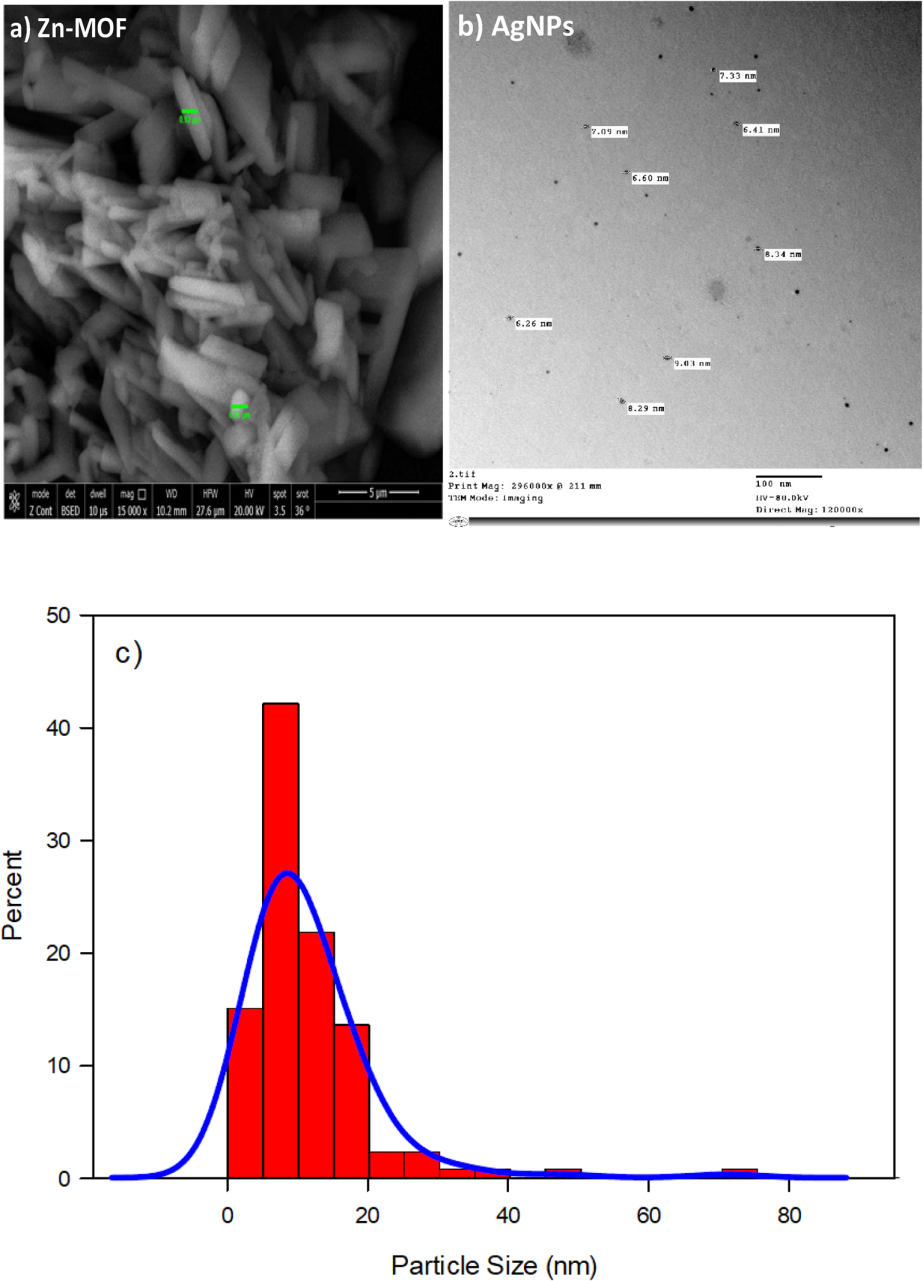
(**a**) SEM image of Zn-MOF, (**b)** TEM image of AgNPs, and (**c**) Particle size distribution of prepared AgNPs.

The morphology, including size and shape, of the **Zn-MOF** material was analyzed using scanning electron microscopy (SEM) as shown in Fig. 5a. The SEM image showed aggregated polygonal plate-like morphology of **Zn-MOF** particles with varying dimensions. On other hand, the size and shape of the silver nanoparticles were analyzed using transmission electron microscopy (TEM) as depicted in Fig. 5b. Understanding the size distribution of particles is particularly crucial, especially for small-sized particles. The particle size distribution was assessed by measuring the diameter of Ag particles utilizing TEM images processed with ImageJ software, as shown in Fig. 5c. Subsequently, the obtained values were depicted in a histogram and fitted with a Gaussian function, revealing a mean particle size of less than 10 nm, as evidenced by the particle size distribution curve. These sizes are significantly smaller than typical bacteria, suggesting their potential efficacy as antimicrobial agents.

**Fig. 6:**
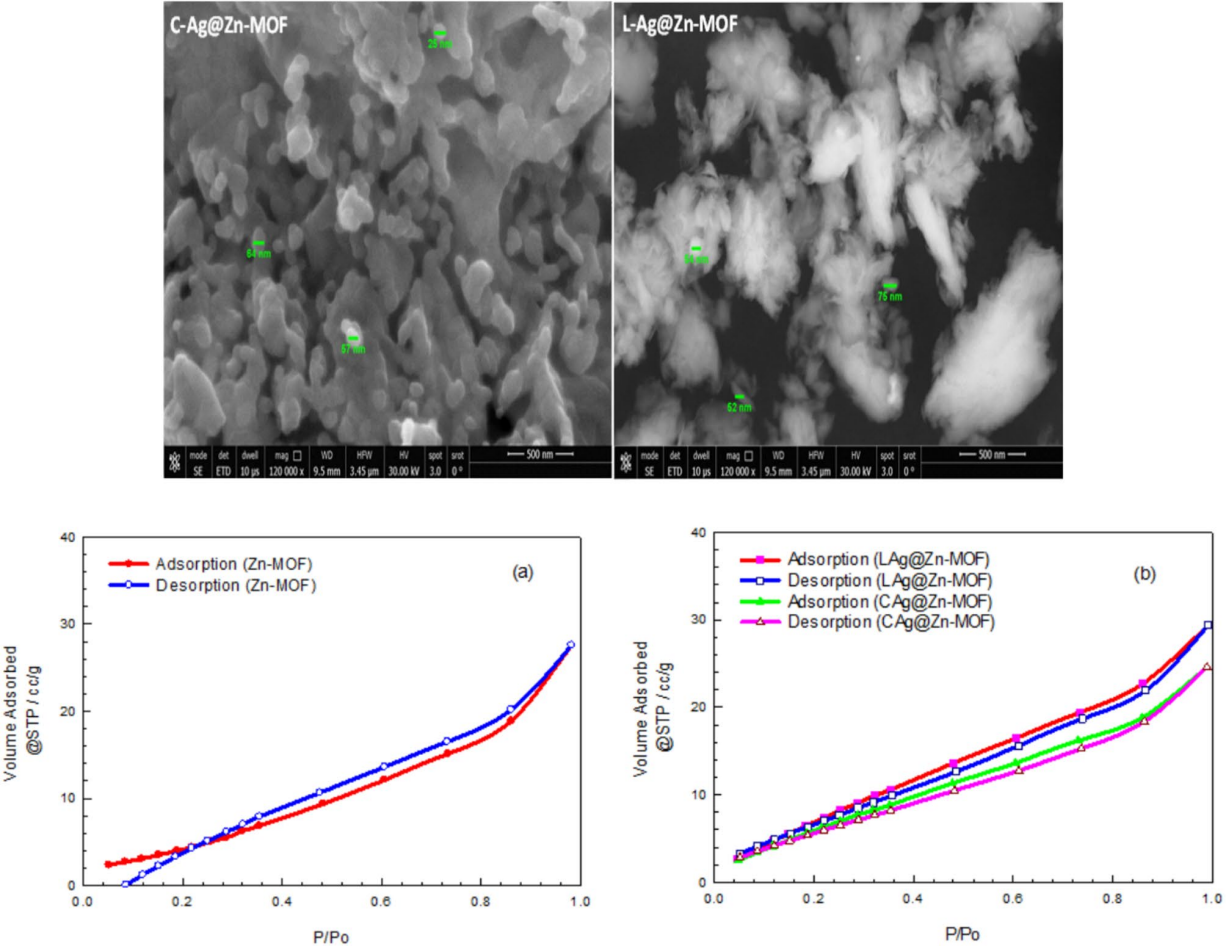
SEM images of Ag/Zn-MOF and Isotherms for Zn-MOF and Ag/Zn-MOF.

In the case of loaded form with AgNPs, the SEM image showed clear accumulation of AgNPs on the surface of the Zn-MOF and this observation was further supported by the measured surface area obtained from N_2_ adsorption–desorption experiment, as seen in Fig. 6. The calculated BET-specific surface area of the modified **Zn-MOF** was higher than that of the pristine **Zn-MOF** indicating an increase in surface area due to the presence of AgNPs (cf. Table 3). However, the average pore size of the modified **Zn-MOF** was found to be lower than that of the pristine **Zn-MOF**. The increase in surface areas of **Ag/Zn-MOF,** particularly in the case of the loaded samples, is significant as it provides more available surface area for contacting microbial species. The increased surface area offers more active sites for adsorption and contact with the investigated microbial species, as compared to the parent **Zn-MOF**^45^.

**Table 3:** Physisorption properties of the Zn-MOF and its hybrid Ag/Zn-MOF.

Compound	Specific surface area (m^2^/g) BET surface area	Porosity volume (cm^3^ g^−1^) Total pore volume	Pore size (nm) Average pore size	Particle radius (nm)
Zn-MOF	20.6705	0.0427709	4.13837	65.970
C-Ag/Zn-MOF	30.2227	0.0381376	2.52377	45.120
L-Ag/Zn-MOF	37.0369	0.0455469	2.45954	36.818

#### DFT study

**Fig. 7:**
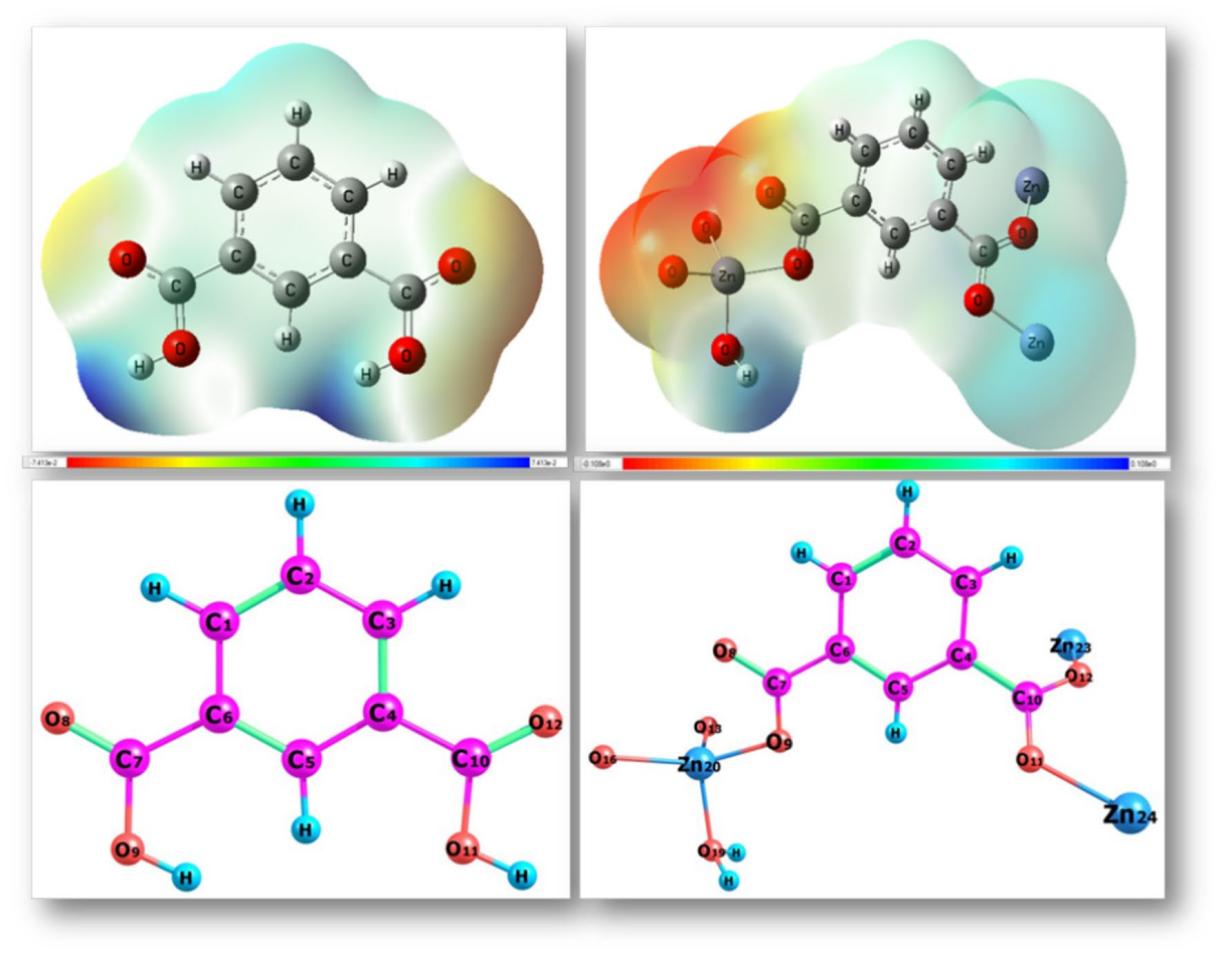
Geometric structure and Mep of IPA linker and Zn-MOF.

**Fig. 8:**
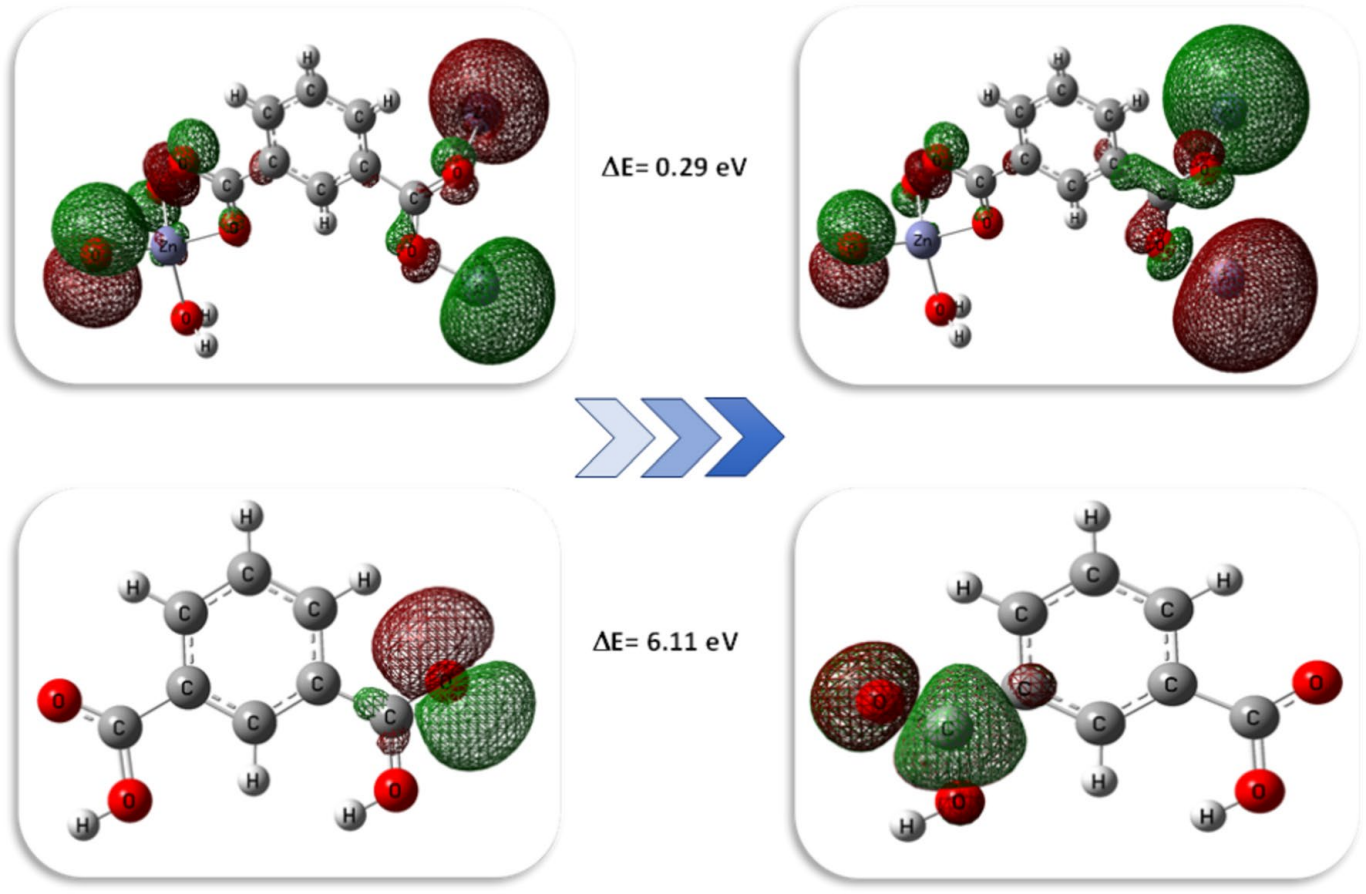
FMO of Zn-MOF and IPA linker, respectively.

The energy value of the prepared **[Zn(IP)(H**_**2**_**O)]** was evaluated using the density functional theory (DFT), with an estimation of several parameters such as dipole moment, hardness (η), softness (S), energy gap, chemical potential (μ) and electronegativity (χ), as mentioned in Table 4. The structure numbering systems and the frontier molecular orbitals (FMO) of the (IPA linker and **[Zn(IP)(H**_**2**_**O)]**) were explored in Figs. 7, 8**.** The DFT analysis revealed that **[Zn(IP)(H**_**2**_**O)]** exhibited more negative values than the starting IPA ligand, indicating a favorable chelation process. The previous parameters were calculated using the following equations:

**Table 4:** Ground state properties and atomic charges in terms of MPA and NPA of IPA linker and its Zn-MOF using B3LYP/6-311G and B3LYP/LANL2DZ respectively.

Parameter	IPA	Zn-MOF (Small unit)	Zn-MOF (Large unit)
E_T_, Hartree	− 609.36	− 1031.39	− 2987.91
E_HOMO_, eV	− 7.56	− 5.96	− 6.23
E_LUMO_, eV	− 1.45	− 5.67	− 6.00
ΔE, eV	6.11	0.29	0.23
I = −E_HOMO_, eV	7.56	5.96	6.23
A =−E _LUMO_, eV	1.45	5.67	6.00
χ, eV	4.5	5.81	6.11
η, eV	3.05	0.15	0.11
S, eV^−1^	0.164	3.42	4.44
µ, eV	− 4.50	− 5.81	− 6.11
Dipole moment (Debye)	3.55	19.27	40.38

$${\varvec{\upeta}}=\frac{(\text{I}-\text{A})}{2}, \mathbf{S} =\frac{1}{2\upeta }, {\varvec{\upmu}} =\frac{-\left(\text{I }+\text{A}\right)}{2}, {\varvec{\upchi}} =\frac{(\text{I }+\text{ A})}{2}.$$

**[Zn(IP)(H**_**2**_**O)]** was found to be a softer molecule than its parent IPA linker, depending on the HOMO-LUMO energy gap. A smaller gap indicates a softer molecule, with the HOMO and LUMO representing the FMO as shown in Fig. 8. The capacitance to accept electrons is called softness and vice versa with hardness^46^.

The energy needed to get rid of the electron is defined by the ionization potential, which equals the HOMO’s energy whereas the LUMO’s energy corresponds to the electron affinity, which produces energy when the system adds one additional electron^47^. A large energy difference was observed between the HOMO energy values of the IPA linker and the zinc-free ion; the linker exhibited a higher LUMO energy (− 1.45 eV) because it acted as the donating source of electrons and vice versa in the case of zinc-free ion (− 12.44 eV). Also, the water molecule, functioning as coordinated ligand, showed analogous behavior to IPA. The electron cloud of the IPA ligand’s HOMO was mainly localized on the carboxylate group. However, in the case of **[Zn(IP)(H**_**2**_**O)],** the zinc ion became included with the carboxylate group. Similar electron density distribution was observed in the case of the LUMO, where both the carboxylate and zinc ion species played crucial roles in the coordination system of **[Zn(IP)(H**_**2**_**O)]**. The negative value for chemical potential and high value for ionization potential contribute to the stability of the compound^48^. The polarity of the IPA linker is increased after binding with zinc ion, as evidenced from the magnitude of their dipole moments^49^.

Changes in bond lengths and angles were observed upon coordination (see Table 5 and Fig. 7), with some bonds elongated and others shortened to achieve the desired tetrahedral structure of the formed **[Zn(IP)(H**_**2**_**O)]**. These observations are due to the formation of the (Zn – O = C) and (Zn – O – C = O) bonds, which make the carbonyl C = O bond weaker and accepted some of the single bond characters^50–53^. The tetrahedral structure was completed with the coordination of water molecule.

**Table 5:** Some of the optimized bond lengths, Å and bond angles, degrees, for IPA ligand and its Zn-MOF using B3LYP/6-311G and B3LYP/LANL2DZ, respectively.

Bond length (A^o^)	IPA	Zn-MOF	Bond angles	IPA	Zn-MOF
R(Zn20-O19(water))	–	1.98368	A(C1-C6-C7)	117.096	119.576
R(Zn20-O9(O-carboxylate))	–	1.95290	A(C6-C7-O8)	123.111	120.342
R(Zn23-O12(C = O- carboxylate))	–	1.97841	A(C6-C7-O9)	118.253	117.804
R(Zn24-O11(O-carboxylate))	–	1.95471	A(C4-C10-O11)	112.908	117.654
R(C1-C2)	1.39357	1.39543	A(C4-C10-O12)	125.502	121.206
R(C2-C3)	1.39451	1.38911	A(O13-O9-O19)	–	62.262
R(C3-C4)	1.40171	1.39070	A(O13-O16-O19)	–	61.646
R(C4-C5)	1.40085	1.38730	A(O9-Zn20-O19)	–	100.726
R(C5-C6)	1.39910	1.39495			
R(C6-C7)	1.49345	1.49510			
R(C7-O8)	1.22746	1.25568			
R(C7-O9)	1.38350	1.26380			
R(C4-C10)	1.47604	1.48521			
R(C10-O11)	1.38303	1.25342			
R(C10-O12)	1.23409	1.26431			

The atomic charges were calculated using Mulliken and Natural Population analysis (MPA and NPA), revealed changes in charge distribution, with some atoms (**O8, O9** and **O11**) exhibiting predominantly negative charges, particularly those involved in coordination with the zinc ion. The molecular electrostatic potential (MEP) analysis indicated specific structural reactive sites, with atoms exhibiting positive electrostatic potential can connect to the electrostatic negative potential sphere, serving as active sites for zinc ion coordination.

Overall, the investigated **[Zn(IP)(H**_**2**_**O)]** exhibited positive electrostatic potential (ESP) maps on their skeletons and negative ESP on their coordination centers, enhancing their antimicrobial activity^54^. The charge of (**O11**) as a representative example was changed from − 0.58 to − 0.46 due to the charge transfer from donating active atom to zinc ion with increasing the electron densities along the zinc ions from + 2 to + 0.519150, + 0.402530 and + 0.945793 as observed in Zn24, Zn23 and Zn20 repeated zinc ions, respectively. The oxygen electron density of the water molecule was decreased upon complexation from − 0.976 to − 0.674 corresponding to the uncoordinated and the coordinated water states respectively, this is due to ligand to zinc charge transfer (L → M), sp^3^ hybridization binding type of the assumed tetrahedral structure. The Atomic charge changes for IPA and its Zn-MOF are tabulated in Table 6.

**Table 6:** Atomic charges in terms of (MPA and NPA) for IPA and its Zn-MOF using B3LYP/6-311G and B3LYP/LANL2DZ, respectively.

Element	IPA MPA(NPA)	Zn-MOF MPA(NPA)
C1	−0.070244 (−0.12200)	− 0.406118 (− 0.09391)
C2	−0.174888 (−0.19407)	− 0.278359 (− 0.15043)
C3	−0.042333 (−0.12508)	− 0.435784 (− 0.15553)
C4	−0.223050 (−0.15749)	0.253206 (− 0.14881)
C5	0.013425 (−0.14467)	− 0.510355 (− 0.11233)
C6	−0.227845 (−0.16505)	0.238346 (− 0.09531)
C7	0.546893 (0.76139)	0.219112 (0.80126)
O8	−0.348180 (−0.53891)	− 0.333986 (− 0.64683)
O9	−0.548719 (−0.66643)	− 0.561299 (− 0.83699)
C10	0.480188 (0.76581)	0.253941 (0.86734)
O11	−0.586750 (−0.69753)	− 0.469121 (− 0.81064)
O12	−0.355473 (−0.57056)	− 0.509452 (− 0.86795)
O13	–	− 0.439358 (− 0.52101)
O16	–	− 0.442012 (− 0.54020)
O19	–	− 0.674868 (− 0.89990)
Zn24		0.519150 (0.66514)

### Molecular dynamics (MD) simulation

**Fig. 9:**
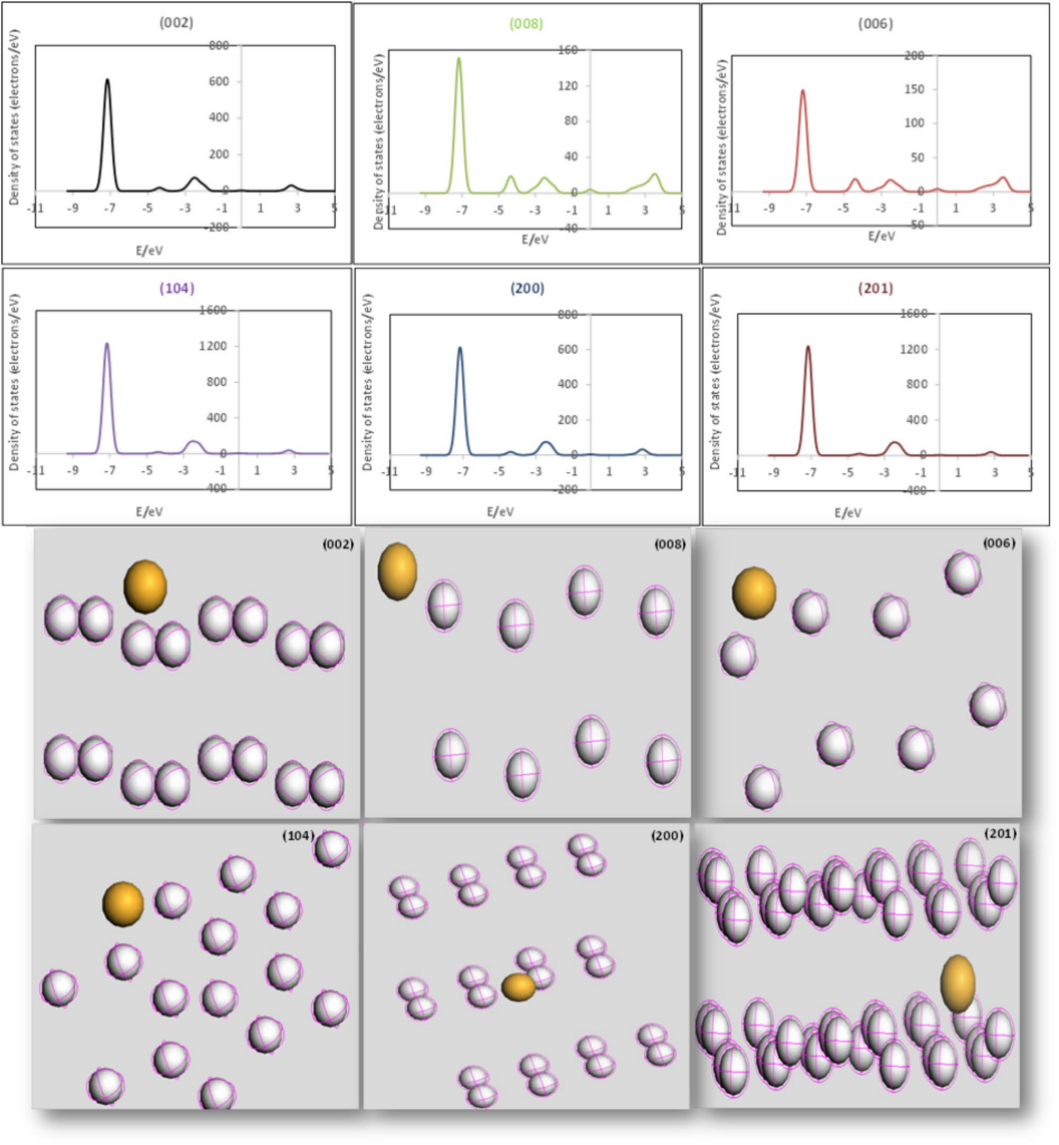
Silver adsorbed on Zn-MOF through different planes with the partial density of state.

The theoretical study was extended to investigate the adsorption of silver atoms on zinc centers of **[Zn(IP)(H**_**2**_**O)]**. Using the material studio program and adsorbate calculation, various planes (hkl) identified from the XRD chart were evaluated for modeling the adsorption process and determining the adsorption energy. Figure 9 and Table 7 represent different planes of **Zn-MOF** with calculated adsorption energy values relative to specific planes. The negative binding energies represent a stronger interaction between silver atoms and the zinc centers. Density of States (DOS) plots were analyzed to assess electronic band parentage, providing information about the type of charge transport that may exist^55^. Figure 9 shows the density of states, represented by the number of electrons. We found the order of electron density to be (104) ~ (201) > (200 ~ 002) > (008) ~ (006), which could be correlated to the number of available zinc atoms serving as the adsorbate surface for the silver atom in each selected plane. Table 7 presents the output and descriptors calculated by Monte Carlo simulation, including parameters such as substrate–adsorbate total energy, deformation energy and rigid adsorption energy. The substrate–adsorbate total energy reflects the sum of deformation and rigid adsorption energies, while the deformation energy reports the energy released when the adsorbate components are relaxed on the substrate surface Rigid adsorption energy refers to the energy released (or required) when the unrelaxed adsorbate components are adsorbed on the substrate.

**Table 7:** The outputs and descriptors were calculated by Monte Carlo simulation for the adsorption of silver on several planes of Zn-MOF.

planes	Total energy kJ mol^−1^	Adsorption energy kJ mol^−1^	Rigid ad. energy kJ mol^−1^	Deformation energy kJ mol^−1^	Atomistic: dEad/dNi
(002)	−0.164	−0.164	−0.164	0.0	−0.164
(006)	−0.164	−0.164	−0.164	0.0	−0.164
(008)	−0.163	−0.163	−0.163	0.0	−0.163
(010)	−0.166	−0.166	−0.166	0.0	−0.166
(012)	−0.164	−0.164	−0.164	0.0	−0.164
(104)	−0.165	−0.165	−0.165	0.0	−0.165
(111)	−0.164	−0.164	−0.164	0.0	−0.164
(200)	−0.165	−0.165	−0.165	0.0	−0.165
(201)	−0.165	−0.165	−0.165	0.0	−0.165
(211)	−0.165	−0.165	−0.165	0.0	−0.165
(217)	−0.161	−0.161	−0.161	0.0	−0.161

### Antibacterial and fungal activity with docking study

#### Agar well diffusion method

**Fig. 10:**
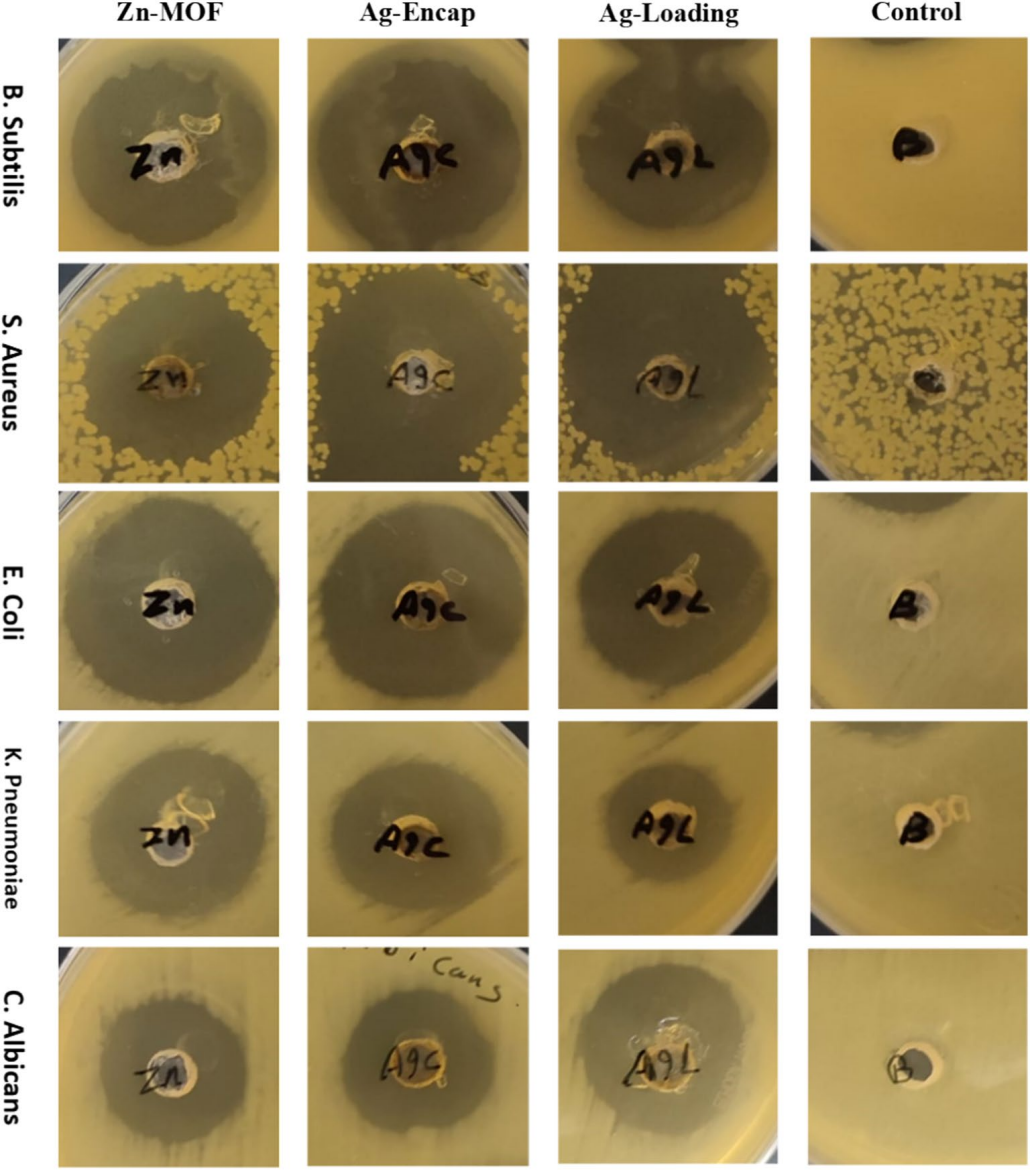
Well diffusion results in agar.

**Fig. 11:**
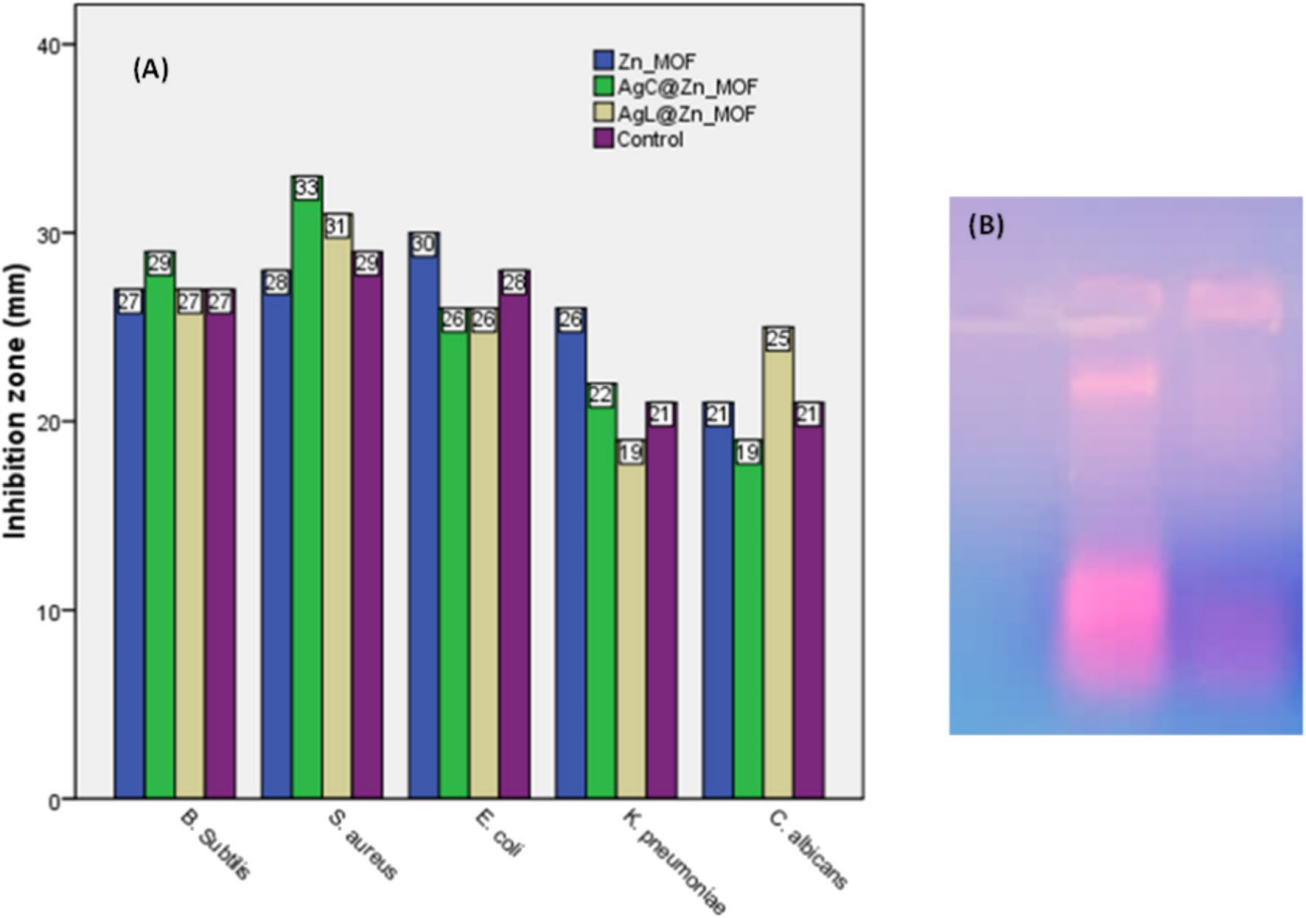
(**A**) Biological activity of Zn-MOF and its hybrid AgNPs towards different types of bacterial and fungal strains; (**B**) The DNA profile damage of *P. aeruginosa* in the absence (control) and presence of the tested drug (10 µg/mL) relative to control using 1% DNA gel electrophoresis.

Evaluation of the antibacterial effects against different bacterial strains was conducted using the agar well diffusion method, with inhibition zones serving as indicators of antimicrobial activity (Figs. 10, 11 and Table 8**)**. The results demonstrated a broad-spectrum inhibition effect against both gram-positive and gram-negative bacterial strains, including *Bacillus Subtilis*, *Staphylococcus aureus, Escherichia coli* and *Pseudomonas aeruginosa*. Remarkably, the synthesized samples exhibited comparable or even superior activity to the standard antibiotic, Gentamicin, against gram-positive bacteria. The enhanced efficacy of the **C-Ag/Zn-MOF** sample, against *B. Subtilis* and *S. aureus,* may be attributed to the combined effects of AgNPs and **[Zn(IP)(H**_**2**_**O)]**. The increased surface area of **Ag/Zn-MOF,** as determined from BET measurements, provides more active sites for interaction with bacterial species. Furthermore, the controlled release of their components from MOF, either as reservoirs or carriers, emphasizes their advantage in antimicrobial applications^56^. Silver nanoparticles, can permeate microbial cell membranes, disrupting DNA and enzyme function, leading to microbial destruction^57,58^.

**Table 8:** Antimicrobial activities data in (mm) of Zn-MOF and its hybrid forms.

Sample; pathogenic microorganism	Zn-MOF	C-Ag/Zn-MOF	L-Ag/Zn-MOF	Control	
*Bacillus Subtilis (ATCC 6633)*	27	29	27	27	**Gentamycin**
*Staph. aureus (ATCC 6538)*	28	33	31	29	
*Escherichia coli (ATCC 8739)*	30	26	26	28	
*K. pneumoniae (ATCC 13883)*	26	22	19	21	
*Candida albicans (ATCC 10221)*	21	19	25	21	**Amphotericin**
*Aspergillus Niger*	NA	NA	NA	18	

*NA* No activity.

Significant values are in bold.

Interestingly, SEM images of **Ag/Zn-MOF** illustrate the adsorption of AgNPs **(**Fig. 6), further supporting the enhanced antibacterial activity observed. Notably, **[Zn(IP)(H**_**2**_**O)]** alone exhibited potent suppression of gram-negative growth, achieving inhibition percentages of 107.14 and 123.8% against *E. coli* & *P. aeruginosa,* respectively relative to the standard antibiotic used. This may be attributed to their highly negatively charged surface, possibly related to the lipopolysaccharides or teichoic acids with phosphate-rich constituents. Such surface characteristics may favor the release of positively charged zinc ion, which could act more effectively against gram-negative bacteria during the examination. However, the surface of AgNPs introduced through loading and encapsulating techniques provided zero-charged Ag particles. This characteristic may result in lower attraction with the negatively charged bacterial surface. Additionally, it may contribute to blocking the release of zinc ions as seen in the morphology structures of **Ag/Zn-MOF** observed in both cases.

Moreover, it was reported that concentrations above 10^−4^ M of Zn^2+^ ions could disturb cellular homeostasis and exert cytotoxic effects on prokaryotes^59^. In general, the varied effectiveness against different bacterial strains is likely influenced by their distinct cellular structures^60^. Unlike *S. aureus*, *E. coli* possesses an additional outer layer of liposaccharides, this structural difference can lead to differences in their functions and responses to antibacterial agents^61^.

Comparative analysis with other Zn-based MOFs revealed the superior antibacterial activity of our synthesized samples. Table 9 illustrates the more favorable effects observed in our samples. For instance, Zn-MOFs based on terephthalate, studied by Akhbari et al., reported a maximum inhibition zone value of 16 mm against *S. aureus* using 5000 μg/mL^1^. Additionally, both Zn-based materials studied by Diéguez et al.^21^ composed of 5-((4-carboxyphenyl)ethynyl), isophthalic acid, and formate anion, achieved significant antibacterial performance against both *S. aureus* and *E. Coli* strains, likely due to more progressive Zn release. Furthermore, Darabpour et al.^23^ discussed the antibacterial results of a Zn-MOF based on 2-Aminoterephthalic acid and 1,4-bis(4-pyridyl)-2,3-diaza-1,3-butadiene, combined with fabricated AgNPs/Zn-MOF nanocomposites achieved a 12 mm against the S. aureus strain at 8 mg/mL of the composite. In conclusion, our study highlights the potential of the synthesized **[Zn(IP)(H**_**2**_**O)]** as an effective antibacterial agent, surpassing standard market drug and similar Zn-MOFs.

**Table 9:** Previously reported Zn-MOFs’ antimicrobial activity summary.

Samples	*S. aureus*	*E. coli*	Concentration	Reference	Note
MOF-5	10	–	50 mg/mL	^1^	Ac; activated^1^ MOF-5 ([Zn_4_O(BDC)_3_].(DMF)_9_), Zn-MOF(A) ([Zn_3_(BDC)_3_(H_2_O)_3_].4DMF) and TMU-3 ([HDMA]_2_[Zn_2_(BDC)_3_(DMA)_2_].6DMF) where BDC: benzene1,4-dicarboxylic acid, HDMA: dimethylammonium and DMA: dimethylamine Zn-MOF(B): 2-Aminoterephthalic acid and 1,4-bis(4-pyridyl)-2,3-diaza-1,3-butadiene linkers
Ac MOF-5	16	10			
Zn-MOF(A)	15.5	14			
Ac Zn-MOF(A)	16	14			
TMU-3	10	10			
Ac TMU-3	14	12			
Zn-MOF(B)	9	R	8 mg/mL	^59^	
Ag/Zn-MOF (1)	12	11	8 mg/mL	^59^	
Ag/Zn-MOF (2)	10	9			
Ag/Zn-MOF (3)	9	R			
Zn-IP MOF	28	30	10 mg/ml	This work	

Fungal species such as *Candida albicans* and *Aspergillus Niger* were tested against the three samples in comparison to the effect of the standard drug amphotericin B. Unfortunately, our compounds exhibited no activity against *Aspergillus Niger*. Nonetheless, the AgNPs loaded sample showed promising results against *C. albicans* outperforming the standard drug, while the parent compound **[Zn(IP)(H**_**2**_**O)]** exhibited similar inhibition activity to the reference drug.

#### Molecular docking

**Fig. 12:**
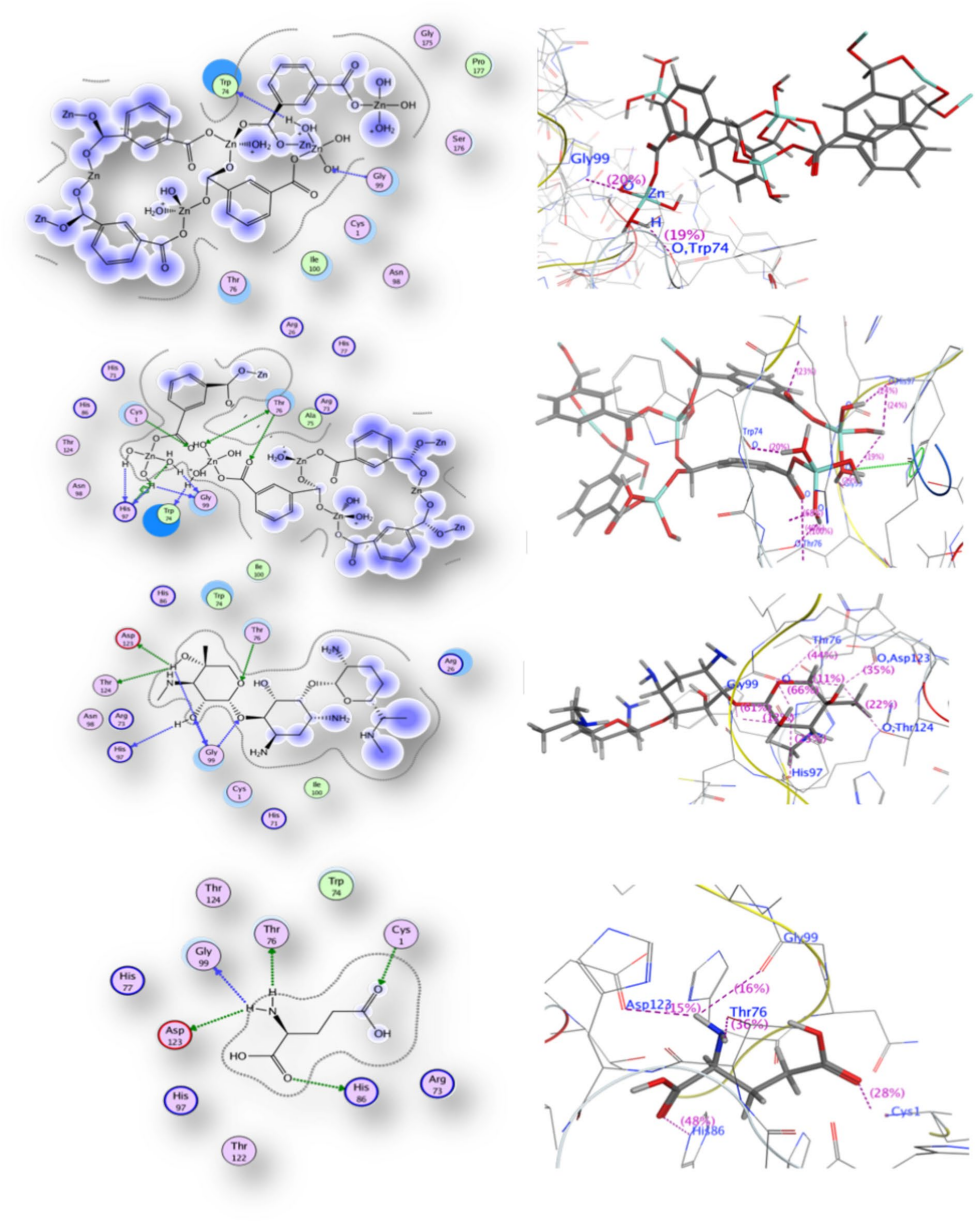
2D and 3D glucosamine-6-phosphate synthase receptor docking results with Zn-MOF, Gentamycin and glutamic acid compounds.

A molecular docking investigation was performed on the prepared **[Zn(IP)(H**_**2**_**O)]** against glucosamine-6-phosphate synthase enzyme (Code:1XFF). Various types of interaction with each amino acid were tabulated in Table 10 and were represented in Fig. 12**,** along with scoring energy and hydrogen bond values. The best-fit sit of enzyme interaction with **[Zn(IP)(H**_**2**_**O)]** revealed a backbone donor interaction between hetero atom source (O) from Gly-*99* amino acid and the Zn-MOF. A backbone acceptor interaction was observed between the water molecule from MOF and the carboxylate group (O) of Trp-74 amino acid; with a hydrogen bond length of 2.08 Å. The scoring energy achieved was − 3.65 kcal/mol supporting the excellent experimental antibacterial results.

**Table 10:** Comparison of binding affinity of Zn-MOF against glucosamine-6-phosphate synthase enzyme using (Code: 1XFF) with root mean square deviation values (RMSD).

Docking 1XFF			
Compound	Scoring energy (kcal/mol) (RMSD)	Involved amino acids (Atom bonding)	Type of interaction (MOF atom bonding)
Zn-MOF (Theoretical fit sit)	−3.65 (3.0)	Gly-*99* and Trp-*74* (2.08 Å)	Backbone donor and backbone acceptor
Zn-MOF (Glutamate sit)	−5.98(1.5)	Thr-*76* (1.88^o^A and 2.47 Å) Gly-*99* (1.49^o^A and 2.78 Å), His-*97* (1.99^o^A and 1.88 Å) (Trp-*74*) (1.63^o^A) Cys-1	Sidechain acceptor and sidechain donor Backbone acceptor and backbone acceptor Backbone acceptor and arene-cation interaction Backbone acceptor water Sidechain donor
Glutamate	−6.93(1.06)	His-*86* (2.94 Å) Asp-*123* (2.31 Å) Gly-*99* (2.86 Å) Thr-*76* (1.96 Å) Cys-*1* (3.0 Å)	Side chain acceptor Side chain acceptor Backbone acceptor amino Side chain acceptor amino Sidechain donor
Gentamycin	−5.04(3.0)	Gly-*99* (1.73 and 3.13 Å) His-*86* (3.58 Å) Thr-*76* (2.93 Å) Thr-*124* (3.07 Å) Asp-*123* (1.97 Å)	Backbone acceptor and donor Backbone acceptor Side chain acceptor Side chain acceptor Side chain acceptor

The MOF docking using the co-crystalized glutamate ligand binding site produces a more effective scoring energy with a more negative value of − 5.98 kcal/mol, indicating favorable interaction. This interaction involved different amino acids such as Thr-*76*, Gly-*99*, His-*97,* Trp-*74* and Cys-*1*. The carbonyl oxygen of the isophthalate linker interacted with Thr-*76* and Cys-1 amino acids, while the water molecule interacted with Gly-*99*, His-*97, and* Trp-74. Water molecule is the main source of hydrogen bond formation with bond lengths mentioned in Table 10. Gentamicin, when docked, exhibited a scoring energy value lower than **[Zn(IP)(H**_**2**_**O)],** consistent with its antibacterial results. Gentamicin interacted with Gly-*99*, His-*86*, Thr-*76*, Thr-*124 and* Asp-*123* amino acids, with OH and -O- functional groups shared from the gentamicin molecule with different amino acids. The docking procedure was validated by re-docking the co-crystalized compound glutamic acid in its binding pocket, revealing a good pose with a scoring energy of − 6.93 kcal/mol as seen in Table 10 and Fig. 12. Similar amino acids were participated in the interactions with **[Zn(IP)(H**_**2**_**O)]**, gentamicin, and glutamic acid.

#### *P. aeruginosa* DNA damaging study

For a comprehensive investigation; the parent compound **[Zn(IP)(H**_**2**_**O)]** was evaluated as a disrupting agent against the bacterial DNA using *P. aeruginosa* DNA as a representative example. The profile was verified by electrophoresis technology using the pure genomic DNA of *P. aeruginosa* pretreated with different concentrations of the tested compound. After a five-hour treatment, the tested **[Zn(IP)(H**_**2**_**O)]** at approximately 10 µg/mL caused fragmentation of *P. aeruginosa* genomic DNA, as observed on the gel **(**Fig. 11B**).** This resulted in smearing, corresponding to degraded genomic DNA compared to the intact one observed in the control (in the absence of **[Zn(IP)(H**_**2**_**O)]**). These findings suggest that the tested compound is strong enough to induce fragmentation of *P. aeruginosa* DNA, highlighting the potential of **Zn-MOF** as an antimicrobial agent. This result confirms the findings of the antimicrobial activity screening.

### Structure–activity relationships of antioxidant and antitumor activity in vitro

Antioxidants perform by inhibiting the effects regulated via free radicals and oxidizing compounds*.* The evaluation of the free radical scavenging activity is a typically performed using DPPH assay, where DPPH molecule is neutralized upon accepting hydrogen or an electron^62^. The scavenging activity percentages of **[Zn(IP)(H**_**2**_**O)]** were calculated at different concentrations and compared to ascorbic acid, a positive standard drug. The IC_50_ value, representing the concentration at which 50% of the DPPH radicals are scavenged, was obtained by plotting the scavenging activity percentages against the different sample concentrations. The loss of the DPPH transition signal intensity in the presence of our suggested antioxidant is directly proportional to the number of protons or electrons accepted. **[Zn(IP)(H**_**2**_**O)]** exhibited an IC_50_ value of 66.07 µg/mL, compared to 55.5 µg/mL for the standard drug Vitamin C.

**Scheme 1:**
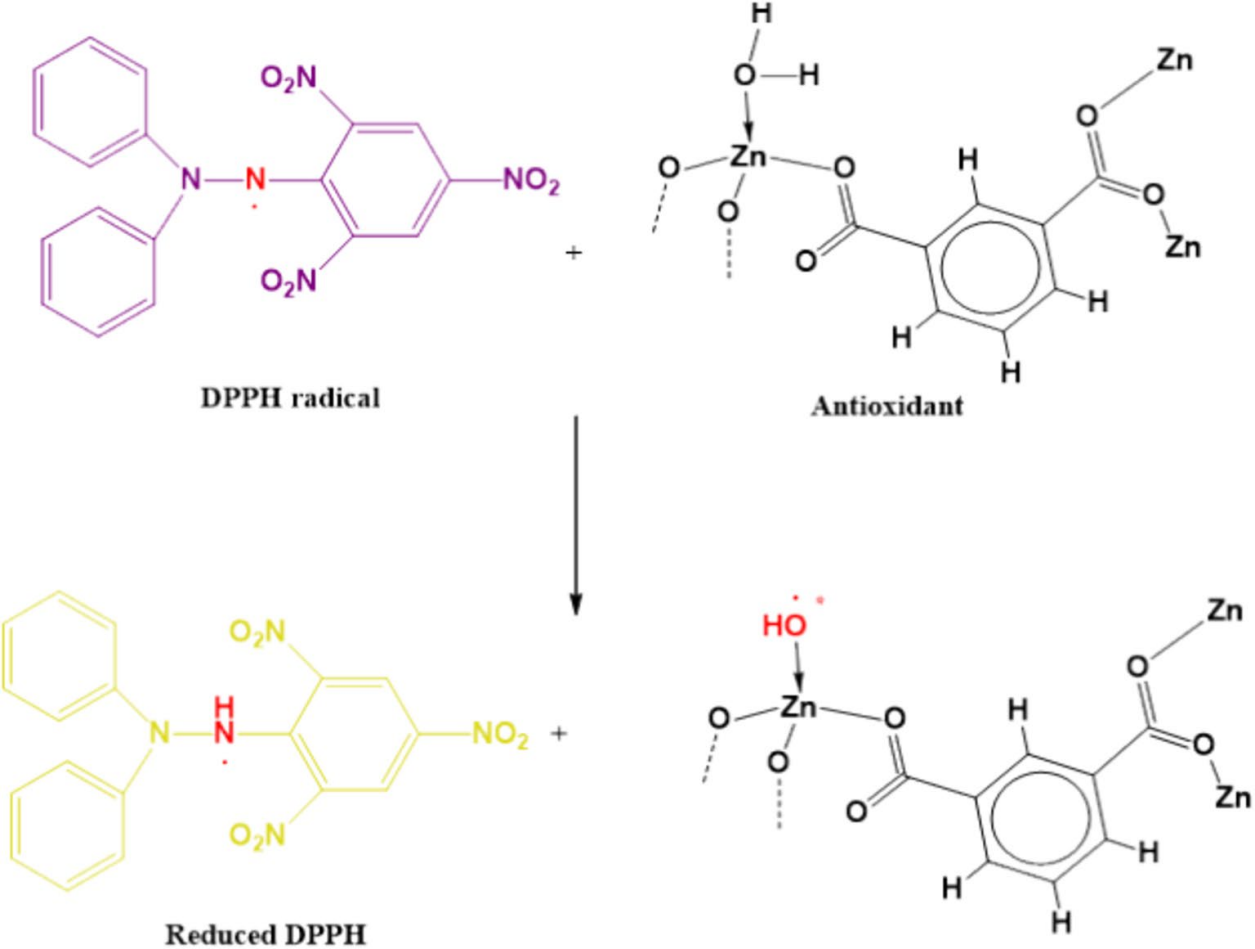
Conversion of DPPH*(purple) to its corresponding hydrazine form (yellow) by the addition of Zn- MOF to DPPH* due to proton transfer.

**Fig. 13:**
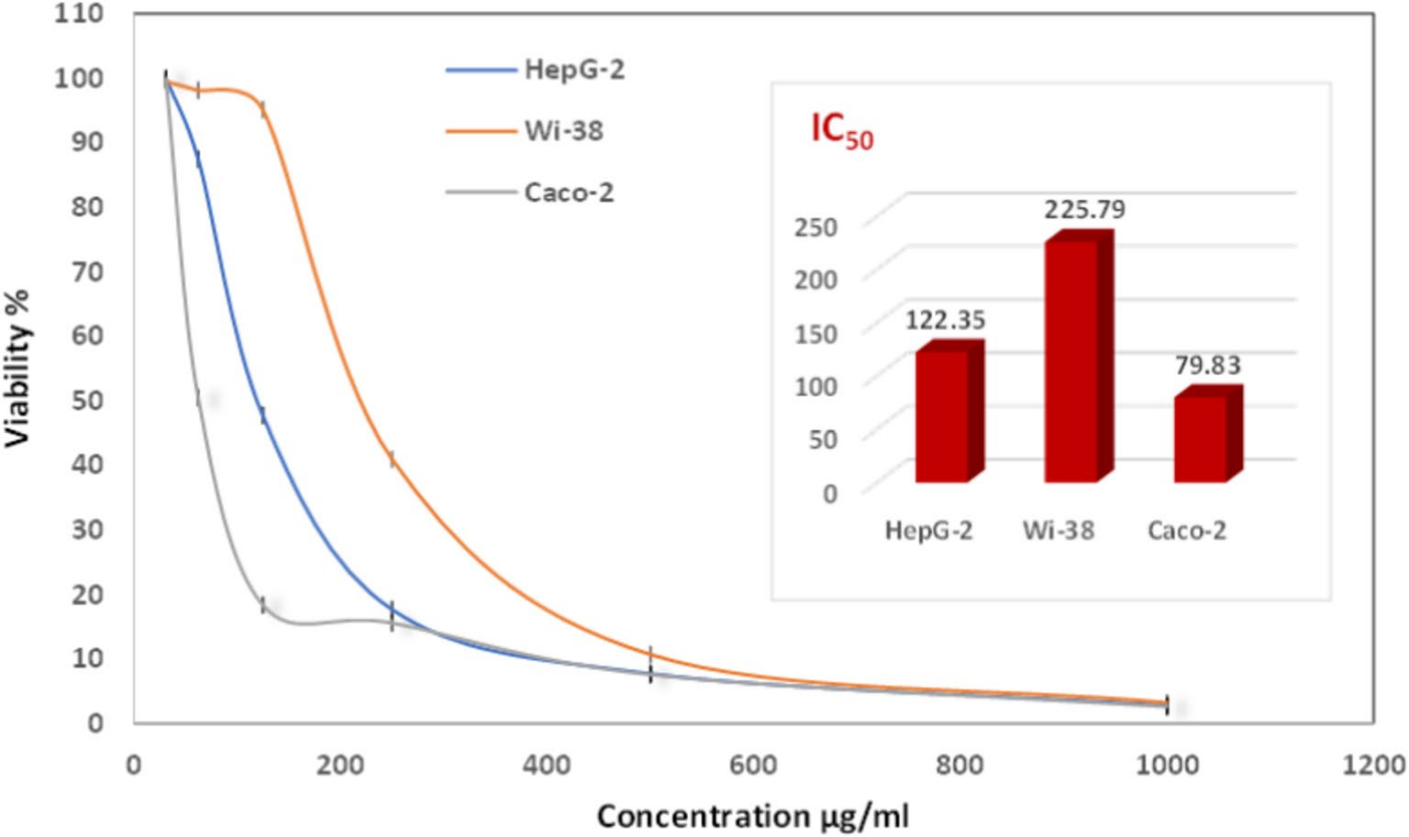
In vitro cytotoxicity of Zn-MOF against the three investigated cell lines with the IC_50_ values.

**Fig. 14:**
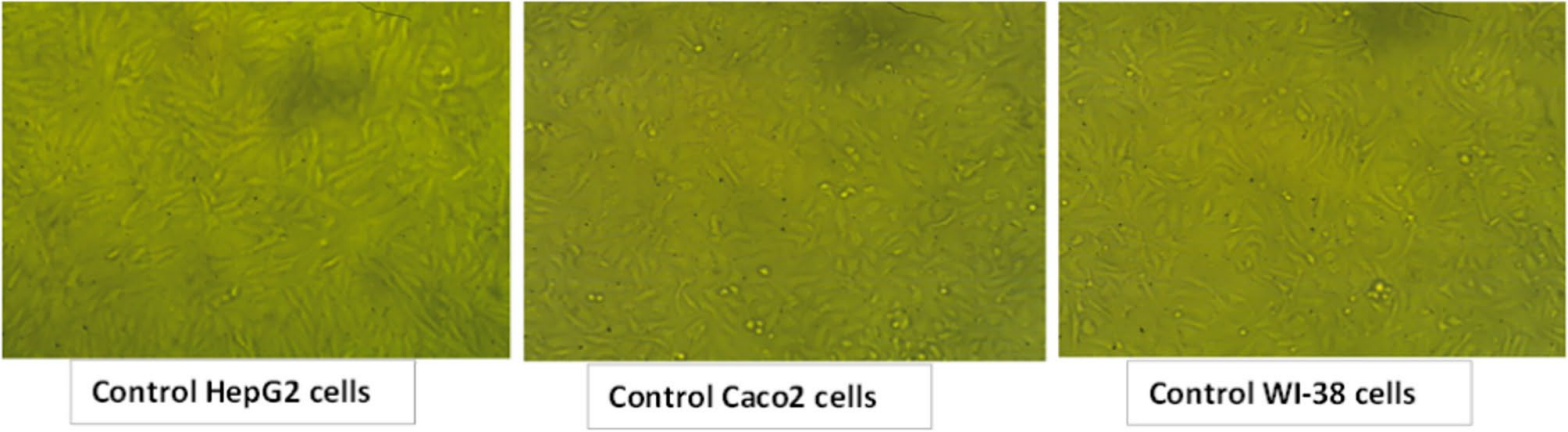
Control cells of the three investigated cell lines.

**Fig. 15:**
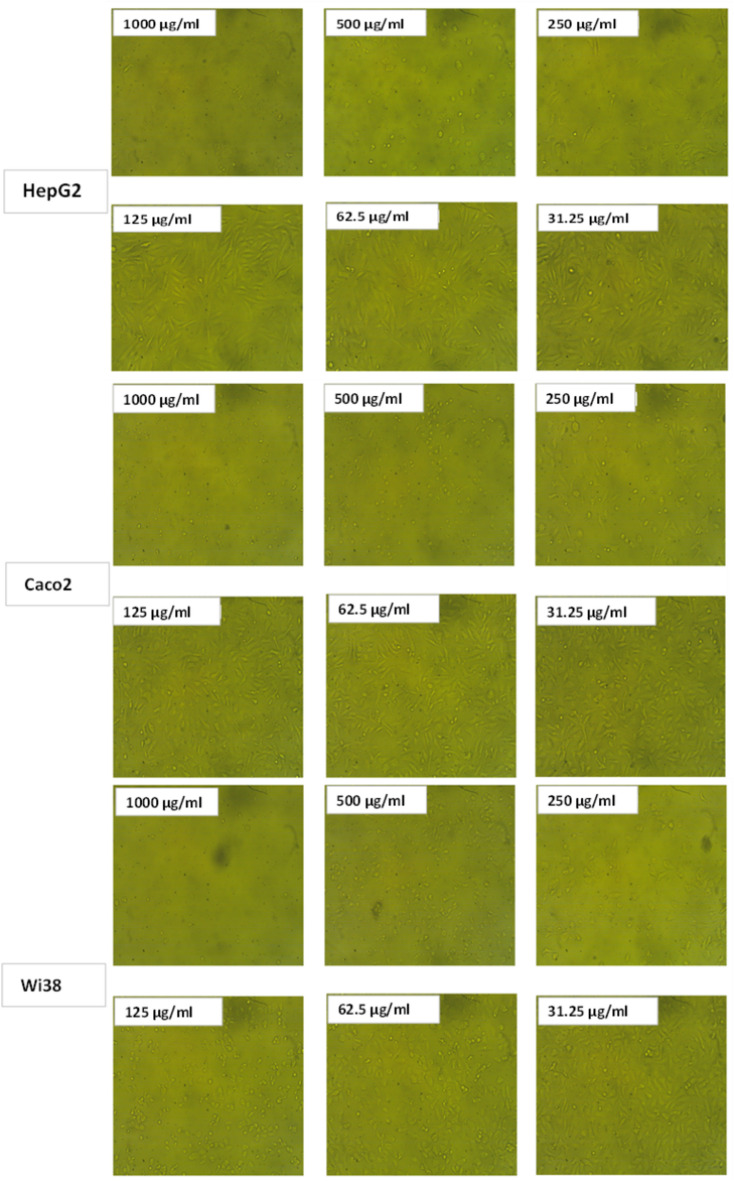
Effect of Zn-MOF on the three investigated cell lines at different concentrations.

Scheme 1 suggests that the coordinated water molecule acts as a proton source due to its induced ionization under the effect of coordination effect, facilitating the ionization of water molecule^63^. Metal chelation is speculated to substantially increase the dissociation constants of the acid substituent in the bound ligand, compared to the free ligand^64^. This interpretation aligns with the high complex formation ability of zinc-chelated compounds, consistent with the Irving–Williams’s order for divalent metals of the 3d series^65^. In *vivo* studies have indicated that the enhanced antioxidant activity of **Zn** compounds may result from the acquisition of additional superoxide dismutating centers, increasing in the molecule’s ability to stabilize unpaired electrons and scavenge free radicals according to Souza et al.^66^. The biological screening was extended to study the potential of **[Zn(IP)(H**_**2**_**O)]** as an antitumor agent. To evaluate the potential of **[Zn(IP)(H**_**2**_**O)]** against colony and liver cancer cells, a standard cell proliferation assay was performed. The inhibition effect versus concentrations is shown in Fig. 13, and images of the cell lines in case of control and treated states with various concentration of **[Zn(IP)(H**_**2**_**O)]** were captured (Figs. 14, 15**)**. The IC_50_ values against human tumor Caco-2 colon and HepG-2 liver cell lines inhibitory concentration which provides 50% killing (Fig. 13**)** were observed, exhibiting earlier achievement of IC_50_ against colon cancer cells compared to liver cancer cells. Specifically, **[Zn(IP)(H**_**2**_**O)]** achieved 50% killing of human tumor Caco-2 colon cell line at an inhibitory concentration of 79.83 µg/mL, with over 80% growth inhibition at 125 µg/mL. In contrast, the IC_50_ value for HepG-2 liver cancer cells was 122.35 µg/mL, with 80.0% inhibition efficiency reached at ≈ 250 µg/mL. Therefore, it seems that the drug resistance in liver cancer cells is higher than the colon cancer cells, or it can expect that the Caco-2 receptors exhibit greater affinity to **[Zn(IP)(H**_**2**_**O)]** compared to the HepG-2 receptors.

It is desirable for a substance to show good cytotoxic activity against various human cancer cells with minimal or no effect on normal cells. So, the study was expanded to investigate the cytotoxic effect of **[Zn(IP)(H**_**2**_**O)]** on a human normal cell line using the human normal lung (Wi-38) cell line. **[Zn(IP)(H**_**2**_**O)]** exhibited minimal adverse effects on normal human lung (Wi-38) tissues. The observed IC_50_ value of 225.79 µg/mL on the Wi-38 cell line suggests minimal activity of **[Zn(IP)(H**_**2**_**O)]** on normal lung cells. Considering that IC_50_ values represent the concentration required for inhibiting cell growth by 50%, the relatively high IC_50_ indicates a lack of significant cytotoxic effect on Wi-38 cells. In comparison, the IC_50_ values for liver and colon cancer cells represent their activation states for exploring cytotoxic effects, indicating a higher sensitivity to **[Zn(IP)(H**_**2**_**O)]**. The calculated inactivation/safety percentages (adverse side effects) are 182.8 and 84.5% for liver and colon cancer cells, respectively, suggesting a strong anticancer effectiveness with minimal adverse effects. Therefore, **[Zn(IP)(H**_**2**_**O)]** shows promise for vivo studies due to its potent anticancer activity and minimal side effects, particularly on sensitive organs like the lungs.

#### Molecular docking

**Fig. 16:**
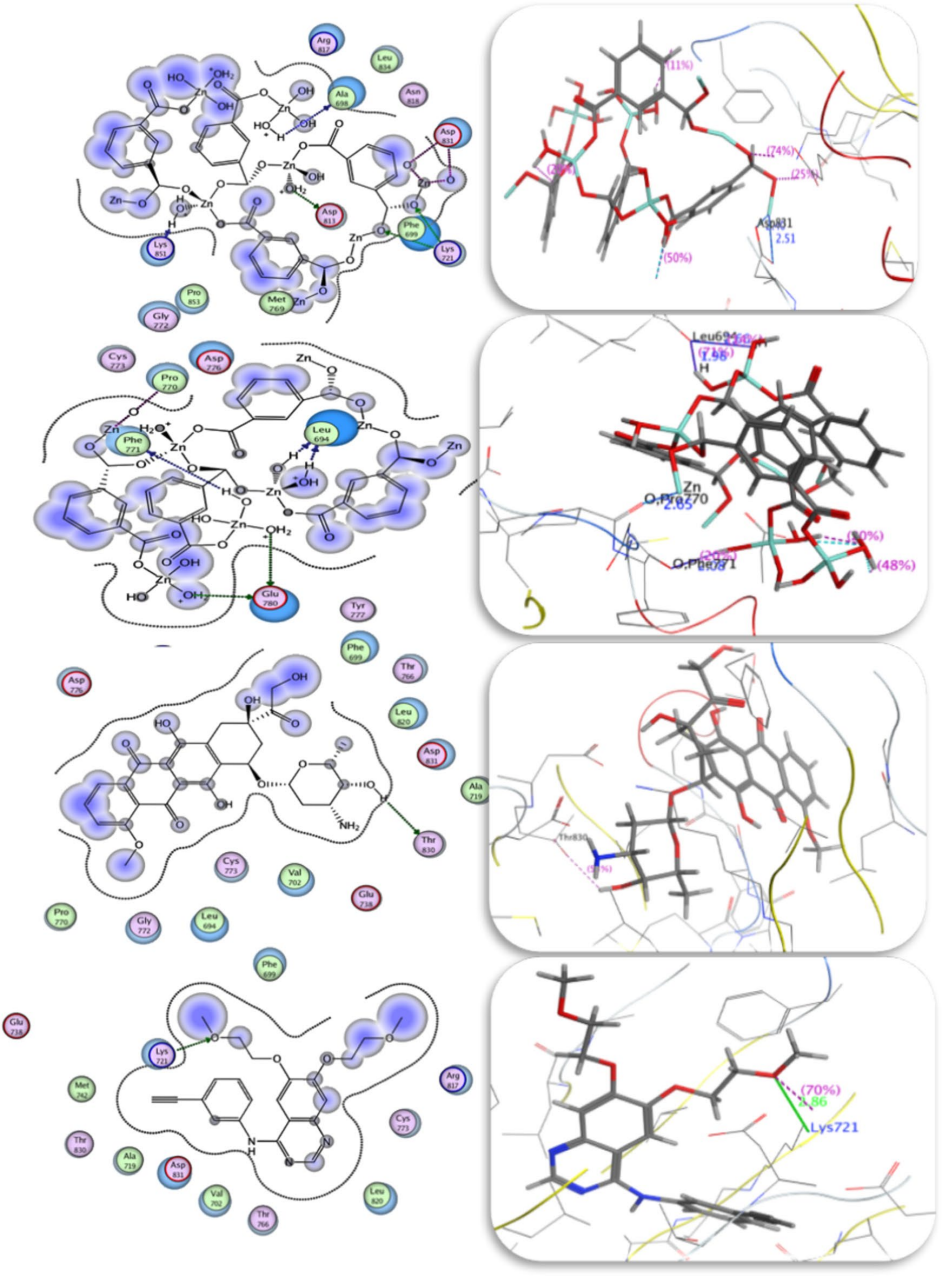
2D and 3D Docking structures Zn-MOF (site finder), Zn-MOF (site AQ4), doxorubicin and AQ4 compounds respectively against EGFR tyrosine kinase (PDB ID: 1M17).

The antitumor docking study results are reported in Fig. 16 and Table 11**.** The most important interaction is the formation of hydrogen bonds, especially those with bond lengths less than 3.0 Å. The most predominant types of interactions observed are the backbone and side chain acceptors. For instance, **[Zn(IP)(H**_**2**_**O)]** formed hydrogen bonds with Phe-*771* (bond length 2.08 Å), while *Glu-780* and pro-770 formed hydrogen bonds with the water molecule of **[Zn(IP)(H**_**2**_**O)]**. The scoring energy value reveals the high binding affinity of **[Zn(IP)(H**_**2**_**O)]** to the selected enzyme. To validate the docking procedure, the co-crystalized ligand in the EGFR tyrosine kinase receptor (PDB Code: 1M17( was re-docked in its binding pocket. The co-crystalized ligand, 4-anilinoquinazoline (AQ4), revealed good poses with RMSD values below 2.0 Å and a high docking score (Table 11**)**. The docking results revealed the interaction between the (N1) quinazoline ring of AQ4 inhibitor and the terminal amino group of Lys-721 amino acid. All bond lengths and amino acids involved in the interactions are tabulated in Table 11.

**Table 11:** Comparison of binding affinity of Zn-MOF against EGFR tyrosine kinase receptor enzyme using (Code: 1m17) with Root Mean Square Deviation values (RMSD).

Docking 1m17			
Compound	Scoring energy (kcal/mol) (RMSD)	Involved amino acids	Type of interaction
Zn-MOF (AQ4 site)	−12.18(2.23)	Phe-*771* (2.08 Å) and Glu*-780* pro-770 (2.65 Å) leu-*694* (2.66 Å, 1.98 Å)	Backbone acceptor and two side chain acceptors Metal contact Backbone acceptor
Zn-MOF (sit finder)	−9.93(2.24)	Lys-*721* Asp-*813* (2.78 Å) Lys-*851* Asp-*831* (2.40 Å and 2.51 Å) *Ala-698*	Side chain donor Side chain acceptor Backbone acceptor Metal contact Backbone acceptor
AQ4	−4.78(2.80)	Lys-*721* (2.86 Å)	Side chain donor
Doxorubicin	−5.45(2.62)	Thr-*830* (2.44 Å)	Side chain acceptor

### DNA binding and cleavage studies

**Fig. 17:**
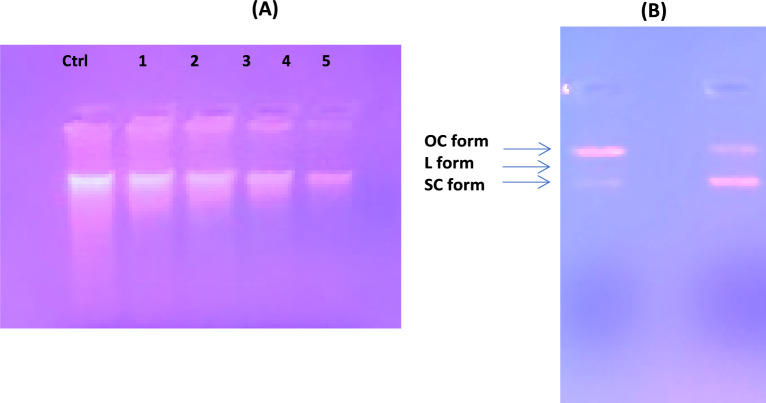
(**A**) DNA electrophoresis of CT-DNA bound Zn-MOF showing different migration and band intensity relative to the free CT-DNA. Where lane M is the unbound CT-DNA (25 µg/ml), while lanes 1–5 are CT-DNA bound to the tested compound of different concentrations (5- 25 µg/ml) respectively; (**B**) PBR322 DNA treated with tested Zn-MOF in comparison to control (absence of the Zn-MOF).

Covalent binding in DNA is irreversible and invariably leads to complete inhibition of DNA processes and subsequent cell death. Cis-platin (cis-diamminedichloroplatinum), a well-known covalent binder used in cancer therapy, forms intra/inter-strand cross-link via its chloro groups with nitrogen on the DNA bases^67^. Therefore, DNA binding was investigated to evaluate the suitability of **[Zn(IP)(H**_**2**_**O)]** as an effective anticancer drug. To evaluate the DNA binding affinity of **[Zn(IP)(H**_**2**_**O)],** different concentrations (5–25 µg/mL) of the **Zn-MOF** were used to monitor electrophoretic mobility and changes in bands intensity of CT-DNA in an agarose gel. The results showed changes in band intensity and migration of CT-DNA induced by binding with the tested compound relative to the control (free CT-DNA) (Fig. 17A). The decrease in electrophoretic mobility of DNA suggests a decrease in total charge and an increase in molecular mass due to binding with **[Zn(IP)(H**_**2**_**O)]** and/or change in the CT-DNA structural conformation^68^. The observed decrease in band intensity (Fig. 17A) with increasing **Zn-MOF** concentration may be attributed to several factors. Firstly, covalent binding of **Zn-MOF** with CT-DNA could damage parts of the DNA, affects its length visualization intensity by ethidium bromide stain^69^. Secondly, the steric effect induced by DNA binding to the metal in **Zn-MOF** may hinder ethidium bromide intercalations. Thirdly, the planar aromatic moiety of the **[Zn(IP)(H**_**2**_**O)]** could intercalate into adjacent DNA pairs, inhibiting ethidium bromide binding to CT-DNA.

The DNA cleavage pattern was further examined using circular DNA plasmid of PBR322. The results indicated that **[Zn(IP)(H**_**2**_**O)]** at 20 μg/mL could convert a relative amount of DNA to the supercoiled form (SC form) while depleting the open circular form (OC form) compared to control plasmid DNA (Fig. 17B). The OC form is produced by scissoring of the SC form^70^. The increase in supercoiling percentage suggests enhanced plasmid quality, which could be beneficial for gene therapy^71^. Therefore, **[Zn(IP)(H**_**2**_**O)]** promise for gene therapy by generating more supercoiled DNA vectors.

### Anti-inflammatory activity

#### Protein denaturation activity

**Fig. 18:**
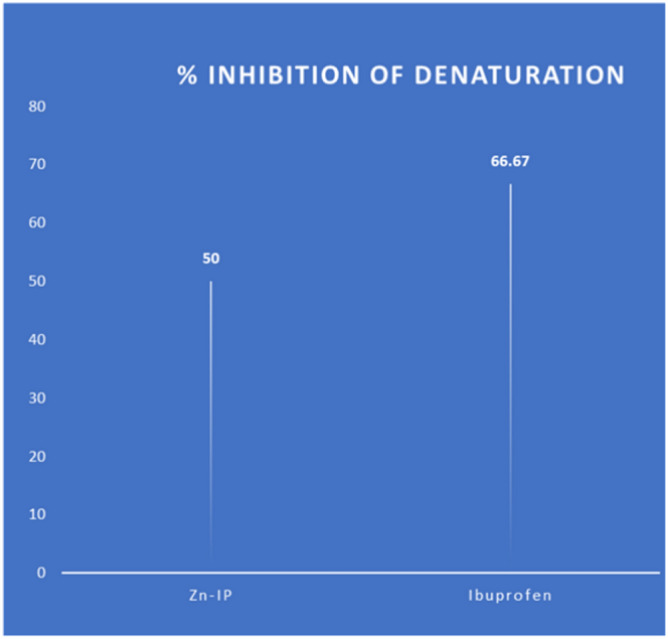
In vitro anti-inflammatory of Zn-MOF and the standard drug.

Non-steroidal anti-inflammatory drugs (NSAIDs) treat inflammation by inhibiting of protein denaturation, inhibiting, hydrolysing enzymes or promoting membrane stabilization. In general, inflammation is caused by either lysosomal enzymes released by leukocytes or protein denaturation as in the case of arthritis^72^. To further explore the pharmacological and biological potential of **[Zn(IP)(H**_**2**_**O)],** its ability to inhibit protein denaturation inhibition was screened. The compound exhibited 50% ± 0.02 inhibition at 10 µg/mL (the obtained result is the mean of three individual measures) indicating its potential as a protein inhibitor and promising anti-inflammatory agent (Fig. 18**)**. This efficacy is comparable to 75.0% efficiency relative to the standard drug ibuprofen. The positive charge of the released zinc ion interacts with BSA which carries a negative charge at physiological pH^73^. Thus, the protein has an overall negative charge (− 18e) at pH 7^74^. Also, the water molecule in **[Zn(IP)(H**_**2**_**O)]** can form hydrogen bonds with BSA. Although further studies are needed to fully understand and confirm the anti-inflammatory activity of **[Zn(IP)(H**_**2**_**O)]**, these initial findings suggest its potential for treating inflammation. Finally, the synergistic effects of its antibacterial and anti-inflammatory properties suggest its potential application as a skin wound healing agent.

## Conclusion

**Zn-MOF** was successfully synthesized using an isophthalic acid linker and a sonochemical method, which proves to be economical, effective and eco-friendly. Analysis techniques revealed that **[Zn(IP)(H**_**2**_**O)]** has a tetrahedral structure, consisting of a Zn atom bonded to three carboxylate groups and one water molecule. The parent **Zn-MOF** and two **Ag/Zn-MOF** samples prepared via loading and encapsulation methods exhibited attractive antimicrobial performance. Furthermore, DNA fragmentation of *P. aeruginosa* supported the efficacy of **Zn-MOF** as an antibacterial agent. **Zn-MOF** demonstrated potential as an efficient anti-inflammatory agent compared to the standard ibuprofen drug. Moreover, it showed promise as an anticancer agent against liver and colon cancer types with minimum adverse effects observed on human normal lungs. The antioxidant efficiency of **Zn-MOF** further supported its anticancer activity. DNA binding studies suggested that **Zn-MOF** could be promising in gene therapy by generating more supercoiled DNA vectors. Computational studies, including DFT and docking, provided insights into the geometric stability of **Zn-MOF** and its interaction with active amino acids in selected proteins associated with the investigated diseases.

## Data Availability

Data available on request with contacting the corresponding author.
